# Quantitative T1 mapping using multi-slice multi-shot inversion recovery EPI

**DOI:** 10.1016/j.neuroimage.2021.117976

**Published:** 2021-07-01

**Authors:** Rosa M. Sanchez Panchuelo, Olivier Mougin, Robert Turner, Susan T. Francis

**Affiliations:** a Sir Peter Mansfield Imaging Centre, School of Physics and Astronomy, University of Nottingham, Nottingham, United Kingdom; b NIHR Nottingham Biomedical Research Centre, University of Nottingham, Nottingham, United Kingdom; c Max Planck Institute for Human Cognitive and Brain Sciences, Leipzig, Germany

**Keywords:** Structural MRI, Quantitative T1 mapping, Multi-slice IR‑EPI, Fat suppression, Neuroanatomy

## Abstract

An efficient multi-slice inversion–recovery EPI (MS-IR-EPI) sequence for fast, high spatial resolution, quantitative T_1_ mapping is presented, using a segmented simultaneous multi-slice acquisition, combined with slice order shifting across multiple acquisitions. The segmented acquisition minimises the effective TE and readout duration compared to a single-shot EPI scheme, reducing geometric distortions to provide high quality T_1_ maps with a narrow point-spread function. The precision and repeatability of MS-IR-EPI T_1_ measurements are assessed using both T_1_-calibrated and T_2_-calibrated ISMRM/NIST phantom spheres at 3 and 7 T and compared with single slice IR and MP2RAGE methods. Magnetization transfer (MT) effects of the spectrally-selective fat-suppression (FS) pulses required for in vivo imaging are shown to shorten the measured *in-vivo* T_1_ values. We model the effect of these fat suppression pulses on T_1_ measurements and show that the model can remove their MT contribution from the measured T_1_, thus providing accurate T_1_ quantification. High spatial resolution T_1_ maps of the human brain generated with MS-IR-EPI at 7 T are compared with those generated with the widely implemented MP2RAGE sequence. Our MS-IR-EPI sequence provides high SNR per unit time and sharper T_1_ maps than MP2RAGE, demonstrating the potential for ultra-high resolution T_1_ mapping and the improved discrimination of functionally relevant cortical areas in the human brain.

## 1 Introduction

Magnetic Resonance relaxometry techniques provide quantitative measures of the longitudinal (T_1_) relaxation time. T_1_ relaxometry removes the confounding effects of sequence-related variations in the signal intensity in T_1_-weighted images – which are also weighted by proton density and T_2_*– facilitating comparison across subjects, scanners and time to characterize the underlying physical composition of tissue, useful clinically in a wide range of applications (for a review, see Cheng et al. (2012)).

The T_1_ relaxation time depends on the concentration of macro-molecules such as proteins, phospholipids, polysaccharides and fat, and the degree of binding of water protons to the macro-molecules, with bound protons having a very short T_1_. Myelin has been shown to be the dominant source of T_1_ contrast in the brain (Koenig et al., 1990; Callaghan et al., 2015; Rooney et al., 2007; Stüber et al., 2014; Leuze et al., 2017), causing T_1_ shortening, although iron also contributes. Stüber et al. (2014) reported that iron makes an average contribution of 10% to T_1_ in white matter and 36% to grey matter in fixed cadaver human brain at 7 T, and Callaghan et al. (2015) showed that myelination is a better predictor of T_1_ than iron content.

Myelin provides the neuronal basis of high processing speeds for high-level cognitive function (see Turner (2019) for a review of the role of myelin in the brain). Quantitative T_1_-maps have been used as a marker of myelination during brain development (Eminian et al., 2018; Kupeli et al., 2020) as well as to characterize white matter demyelination in clinical conditions (e.g. multiple sclerosis (Al-Radaideh et al., 2015). The study of intracortical demyelination patterns is clinically relevant (Mougin et al., 2016; Beck et al., 2018) but still remains a challenge.

In systems neuroscience, quantitative T_1_ maps can provide the basis for *in-vivo* cortical parcellation of distinct cortical areas (Turner, 2019). Ultra-high resolution structural MRI data can depict submillimeter-scale variations in image intensity across the cortical thickness, corresponding to variations in myelin density (e.g. Geyer et al., 2011; Glasser et al., 2016; Mougin et al., 2013; Sánchez-Panchuelo et al., 2012a; Sereno et al., 2013; Turner and Geyer, 2014); for a review, see (Trampel et al., 2019). This intracortical MRI contrast arises almost entirely from myeloarchitecture, rather than cytoarchitecture, although there may often be correspondence between the two (Dinse et al., 2015). T_1_ maps closely resemble myelin-stained histological sections (Geyer et al., 2011). Other studies have highlighted the correlation of observed cortical T_1_ contrast with known distributions of myelination (Glasser and van Essen, 2011; Lutti et al., 2014), paving the way for histological cortical parcellations *in-vivo*. Dinse et al. (2015) showed that a model based on archival cytoarchitectural data from cadaver brain could predict the cortical profile of *in-vivo* quantitative values of T_1,_ bridging the gap between the microanatomy revealed by classical histology and the macroanatomy visible in MRI. This work advocated a spatial resolution of 0.3 mm to reliably distinguish cortical areas based on intracortical features. However, this spatial resolution is beyond the limit of current T_1_ mapping methods *in-vivo* at ultra-high field (UHF, 7 T) and improvements in image acquisition are required to provide adequate ultra-high resolution quantitative T_1_ maps with a narrow point-spread function, maximal SNR per unit time, and low SAR.

A number of techniques have been proposed for T_1_ mapping. The inversion recovery (IR) method is considered the most accurate approach to measure T_1_, acquiring multiple lines of k-space or images at a range of values of the time TI after a 180° inversion in order to sample the longitudinal magnetization recovery curve and derive an estimate of T_1_ by fitting the data to a mono-exponential function. However, the main disadvantage of this method is its temporal inefficiency. The gold standard sequence combines an IR with a spin-echo readout of a single line of k-space to limit the effect of B_1_ and B_0_ inhomogeneities, but the long acquisition time makes this impractical for human studies. Alternatively, the 2D approach can use an echo-planar imaging (EPI) readout to collect an image or series of multi-slice images after a given TI. While this significantly reduces the scan duration, spatial distortions can occur which are intrinsic to the use of single-shot EPI at high spatial resolution. Several methods have been proposed to improve the efficiency of T_1_ mapping including Look-Locker (LL) (Look and Locker, 1970) methods combined with FLASH (Deichmann et al., 1999) or EPI (Gowland and Mansfield, 1993) readouts which estimate T_1_ by collecting multiple read-outs to sample the longitudinal magnetization following a single T_1_-recovery curve. However, the acquisition time remains long as the sequence must be repeated for multiple slices, although simultaneous multi-slice (SMS) strategies can now accelerate such acquisitions. A faster multi-slice inversion recovery variant was introduced (Ordidge et al., 1990) to allow the acquisition of quantitative *T*_1_ maps with whole brain coverage in 3 min. This sequence consists of a spatially non-selective adiabatic inversion pulse followed by the sequential acquisition of multiple slices with single-shot 2D EPI readouts, resulting in each slice being collected at a different inversion time. The slice ordering is then shifted in the next repeat of the data acquisition so that multiple points across the T_1_-recovery curve are collected for each slice (Ordidge et al., 1990; Clare and Jezzard, 2001). If the total number of acquisitions repeated is less than the number of slices, then the computation of T_1_ maps requires the use of slice-specific inversion times, a method which has been shown to produce consistent T_1_ maps across a volume of 2D slices in a minimal acquisition time (Cohen and Polimeni, 2018; Lauzon et al., 2016; Polders et al., 2012; Renvall et al., 2016).

However, T_1_ maps collected using single shot EPI are prone to geometric distortions due to the low bandwidth in the phase-encode direction and B_0_-field inhomogeneity. The MS-IR-EPI work presented in this manuscript aims to provide an alternative to the FLASH-based sequences for 'distortion-free' T_1_ mapping; however it should be noted that for some fMRI applications single-shot IR-EPI T_1_ maps are collected by design and intent to provide anatomical data with matched distortions to the fMRI data (Renvall et al., 2016). Such single-shot EPI T_1_ maps can be acquired with submillimeter (0.7 mm) spatial resolution over a small field of view to improve the accuracy of cortical depth definitions in fMRI data space (Huber et al., 2016; Kashyap et al., 2018). However, for the purpose of systematic cortical parcellation, a higher nominal spatial resolution (0.3 mm) is required over larger brain volumes. For this to be achieved the readout window and effective echo time of single-shot EPI would become prohibitively long, with consequent unacceptable image blurring.

Currently, the most popular method of T_1_ mapping used at UHF is the ‘Magnetization-Prepared 2 RApid Gradient Echoes' (MP2RAGE)’ sequence (Van de Moortele et al., 2009; Marques et al., 2010). This combines two Gradient-Recalled Echo (GRE) 3D volumes acquired at two different inversion times (the first predominantly T_1_-weighted and the second PD-weighted) within a single repetition time to calculate a quantitative T_1_ map based on the T_1_-weighted combination image, which is free from bias-field effects, and the use of a look-up table. A non-selective adiabatic inversion pulse is used to minimize sensitivity to the inhomogeneity of transmit B_1_ field (B_1_^+^) at 7 T (Hurley et al., 2010). Deviation of the nominal excitation flip angles in the GRE trains caused by inhomogeneity in B_1_^+^ can lead to bias in the T_1_ estimation, particularly when a large number of low flip angle excitation pulses are used (Marques and Gruetter, 2013) as required for submillimeter spatial resolution. The further application of a B_1_-mapping sequence can provide estimates of the effective excitation flip angle and thus improve the accuracy of MP2RAGE based T_1_ mapping (Marques and Gruetter, 2013; Haast et al., 2018), enabling measurement of cortical thickness using quantitative T_1_ maps (Haast et al., 2018). However, for ultra-high spatial resolution measures the resulting 3D GRE train is long, of the order of tissue T_1_, resulting in a relatively broad point spread function (PSF) and long total scan duration for sufficient SNR. When MP2RAGE is used at nominal spatial resolution of the order of 0.3 mm to depict intracortical details, these invariably appear blurred, even on trained subjects when no apparent motion is detected or when motion is prospectively corrected (Federau and Gallichan, 2016).

In this present study, we aimed to implement a fast multi-slice multi-shot inversion-recovery 2D-EPI T_1_ mapping method which fills all the recovery time between each inversion pulse with acquisition of k-space segments of each slice, keeping the effective TE and acquisition time to a minimum to reduce geometric distortions, and resulting in a narrow point-spread function. It has been shown that the value of T_1_ in the brain is largely determined by magnetization transfer (MT) between the free water proton pool and the macromolecule-bonded proton pool (Koenig et al., 1990; Turner et al., 2008; Shin et al., 2009). The feature of short-TE EPI sequences which contributes most to magnetization transfer effects is the fat suppression pulse that normally accompanies each spin excitation pulse. To explore the possibility of accurate T_1_ quantification, we assessed the impact of MT effects due to multi-slice acquisitions and the spectrally selective fat suppression pulses on T_1_ quantification that we employed. We propose a model to remove the MT contribution from the derived T_1_. The approach is combined with simultaneous multi-slice (SMS) acquisition techniques to improve spatial coverage.

We hypothesise that: (a) MS-IR-EPI produces T_1_ maps at 7 T with accuracy and repeatability comparable to MP2RAGE and multi-shot single slice IR-EPI, but in a faster time; (b) MS-IR-EPI provides T_1_ maps with higher SNR per unit time than MP2RAGE; (c) the MT effects due to spectrally selective fat suppression pulses in MS-IR-EPI can be effectively removed for accurate T_1_ quantification; (d) MS-IR-EPI T_1_ maps yield sharper images than MP2RAGE with the same nominal spatial resolution, and can be used for intracortical mapping.

## 2 Methods

### 2.1 Multi-shot inversion-recovery 2D echo-planar imaging (MS-IR-EPI) sequence

**Fig. 1:**
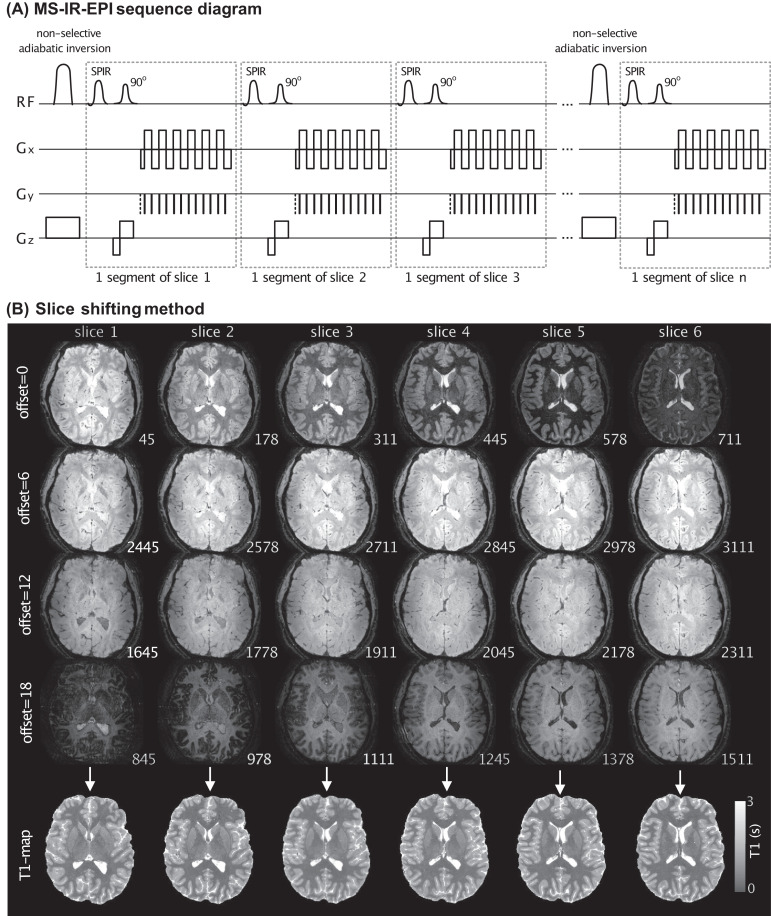
(A) 2D multi-shot inversion recovery EPI sequence diagram: A non-selective adiabatic inversion pulse of slab thickness 200 mm is rapidly followed by a series of multi-slice-selective 90° excitation pulses (spectrally selective SPIR pulses applied for fat suppression). A segment of k-space is acquired after each excitation in order to keep the acquisition window short, to minimize image distortion and dropout and ensure a well-defined inversion time and a narrow point-spread function. In order to generate T_1_ maps, multiple acquisitions are performed with varying slice acquisition order by adding a slice offset to the ascending slice order. (B) Example sub-set of slices (6 of 24) acquired using 2D MS-IR-EPI (0.5 × 0.5 × 1.5mm^3^, TR=3.2 s). Data is shown for four slice offsets of [0,6,12,18] (each row). The different contrast across the slices reflects the different acquired TIs (ms); Note that for each slice the 4 TIs are equally spaced at 800 ms intervals across offsets. The corresponding T_1_ map generated for each slice is show in the bottom row.

Fig. 1 illustrates the multi-shot multi-slice inversion-recovery EPI (MS-IR-EPI) sequence that was implemented. A non-selective adiabatic inversion is used for homogeneous inversion across the imaging volume. The time between successive inversions is filled with slice-selective 90° excitation pulses, each followed by a segmented gradient echo (GE) EPI acquisition window. The segmented acquisition, whose duration is a small fraction of T_1_, is used to provide a well-defined inversion time for each segment and consequently a narrow point-spread function, while multiple shots are required to sample the entire k-space plane. This sequence can be used with in-plane acceleration and SMS excitation pulses to achieve whole brain coverage even more quickly (Dougherty et al., 2014; Grinstead et al., 2014). Multiple volume acquisitions (N_acq_) are collected in which the slice order is shifted by a constant offset number, resulting in a set of equally spaced distinct inversion times for each segment (shown in Fig. 1 for 800 ms temporal slice offset). The slice acquisitions are equally distributed within the TR period after the minimum inversion time (~44.5 ms), and there is no dead time during the recovery of longitudinal magnetization. Note, in contrast to single-shot IR-EPI, the recovery time remains constant across all segments of the multi-shot acquisition, except for when transitioning between slice offset number. The collection of complex data allows the phase data to be employed to correct the polarity in the slice-by-slice computation of T_1_ maps using the modulus corrected data, resulting in a mono-exponential T_1_-recovery curve: S(TI) = S_0_[1–2exp(−TI/T_1_)+exp(-TR/T_1_)], which can easily be fit using a least squares regression. The S_0_ image provided by the fit represents the proton density weighted by the effective transverse relaxation time T_2_*.

### 2.2 Monte Carlo simulations to optimize sampling for the MS-IR‑*EPI* sequence

**Fig. 2:**
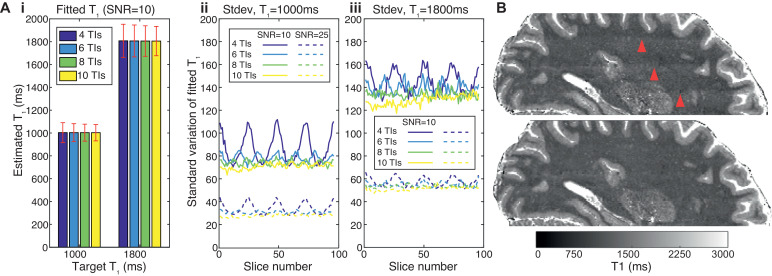
(A) Monte Carlo simulations of the multi-shot inversion recovery EPI sequence showing the effect of number of acquisition/TIs (4,6,8,10) on fitted T_1_ parameters. Simulations were performed for a target T_1_ of 1 s (~WM) and 1.8 s (~GM), SNR=10 and 25, TR=5 s and 96 slices. Gaussian noise was added to the simulated complex data (with 1024 repeats) which was fit after sign correction, as for the experimental data, to obtain mean and standard deviation of fitted T_1_ values. Fig. 2Ai). The fitted T_1_ (mean across slices) remains constant but the standard deviation (error bars) decreases with increasing number of TIs (shown for SNR=10). Fig. 2A ii) plots the standard deviation of the fitted T_1_-values across each of 96 slices for SNR=10 (solid lines) and SNR=25 (dash lines) showing that for WM there is a variation of the fitted T_1_ value across slices when using 4 and 6 TIs whereas in Fig. 2A iii) for GM the variation is only obvious when using only 4 TIs. (B) Sagittal reconstructions of the axial T_1_-maps derived using 4 (top) and 8 (bottom) equally spaced TIs. Note the T_1_maps derived from 4 TI data show banding within the WM tissue (red arrowheads) not evident in the T_1_ map derived from 8 TIs, in agreement with simulations. (For interpretation of the references to colour in this figure legend, the reader is referred to the web version of this article.)

Since the MS-IR-EPI method computes a T_1_ map from the different inversion times experienced by each slice, care must be taken to ensure that the sampling of the inversion times across all slices provides consistent T_1_ values. The minimum TI each slice experiences has a key impact on the measured T_1_, especially for lower T_1_ values, and this can lead to banding artefacts observed in the slice direction when an insufficient number of acquisitions are performed (and hence the minimum TI values sampled). To test the robustness of the T_1_ fitting to the range of acquisition strategy and noise level, a Monte Carlo (MC) simulation of the entire fitting algorithm (including the phase correction) was implemented. Each MC run included 1024 samples with gaussian distributed noise added to the complex signal, at SNR levels of 10 and 25, and target T1s of 1000 and 1800 ms. The MC simulation was used to determine the minimum number of acquisitions (N_acq_ and thus inversion times) needed to obtain homogenous T_1_ maps across the multiple slices of the data set (Fig. 2 and Supplementary Material Figure 1). We assessed N_acq_ for whole brain 0.7 mm isotropic resolution at 7 T (for a TR of 5 and 3.2 s with SMS factors of 2 and 3 respectively), and whole brain 1 mm isotropic resolution at 3 T (for a TR of 4.5 s with SMS factor 2) (See Table 1 and ‘T_1_ mapping data acquisition’ section).

**Table 1:** Parameters used in MS-IR-EPI and MP2RAGE protocols at 7 T and 3 T. For MS-IR-EPI, BW provided in frequency (FE) and phase (PE) encoding direction, SAR provided for SMS option (if used). The SAR for MS1 in the absence of fat suppression was <1.1 kg/W.

MS-IR-EPI																						
Protocol	B_0_	Voxel (mm^3^)	TR (s)		EPI factor		BW_FE/PE_ (Hz)		TE (ms)		SENSE factor		SMS factor		SPIR FA(^o^)		SAR (W/kg)		Slice offsets			Acquisition time
MS1	7T	0.7^3^	5		15		856/61		16		2		1&2		70		<1.6		0,12,24,36,48,60,72,84			4 min 50s
MS2	7T	0.7^3^	3.2		13		884/74		16		1.6		1&3		0		<1.3		0,13, 26, 39, 52			2 min 56s
MS3	7T	0.5^3^	3.8		7		625/104		14		1.6		3		90		<2.8		0, 8, 16, 24, 32, 40, 48, 56, 64, 72			18 min 22s
MS4	7T	0.35^2^×0.7	3.2		13		492/41		20		2		1		60		<1.1		[0, 7, 14, 21, 28]x3			11 min 8s
MS5	3T	1^3^	4.5		11		951/95		11		3		2		0		<0.1		0,12,24,36,48,60,72,84			3 min 54s
**MP2RAGE**																						
Protocol	B_0_	Voxel (mm^3^)		TR_shot_ (s)		T1_1_ (ms)		T1_2_ (ms)		TE/TR (ms)		TFE factor		FA_1_/ FA_2_		SENSE factor		TFE_acq_ (ms)		BW (Hz)	SAR (W/kg)	Acquisition time
MP1	7T	0.7^3^		5		900		2757		2.5/5.5		224		5/3 ^o^		3		1230		337	<0.9	8 min 55s
MP2	7T	0.35^2^×0.7		4		900		2375		9/18		70		5/2 ^o^		2		1277		67.2	<1.1	12 min 8s
MP3	3T	1^3^		5.5		700		2500		3/7.1		118		5/3 ^o^		3 × 1.5		838		228	<0.1	7 min 10s

### 2.3 T_1_ mapping acquisitions

Scanning was performed on a 7 T Philips Achieva system and 3 T Philips Ingenia system, both equipped with a 32-channel receive array NOVA coil. The 7 T whole head MS-IR-EPI protocol comprised a field of view (FOV) of 192(AP) x 164(RL) mm^2^ with 0.7 mm isotropic resolution using protocols MS1 and MS2 outlined in Table 1 with SMS factors of 2 and 3 respectively.

At 3 T, 1 mm isotropic MS-IR-EPI data with whole brain coverage (192 × 192 × 192 mm^3^) were acquired using protocol MS5 parameters with a SMS factor of 2 (Table 1). All MS-IR-EPI protocols acquired contiguous slices in ascending order with phase encoding in the RL direction. The maximum EPI factor used was 15 (see Table 1) to provide T_1_ maps with minimal distortion (see Fig. 5, Sanchez Panchuelo et al., 2018). This factor was lowered in some protocols in order to increase the number of slices required to achieve whole brain coverage (for the specific TR and SMS factor). For comparison to the MS-IR-EPI method, MP2RAGE data with 0.7 mm and 1 mm isotropic resolution were acquired at 7 and 3 T respectively (see Table 1: MP1 (parameters based on Dinse et al. (2015)) and MP3 protocols). A TR-FOCI pulse was used for adiabatic inversion at 7 T (see Hurley et al. (2010) for details of the TR-FOCI pulse). MP2RAGE acquisitions were followed by a DREAM B_1_-map acquisition (4.5 mm isotropic resolution, FOV=288(AP)x252(RL)x198(FH) mm^3^, SENSE factor=2 (RL), FA=8°, TE=1 ms, TR=2.3 ms, Turbo-Field-Echo factor=890, acquisition bandwidth=5351 Hz, 2.2 s acquisition time) to allow computation of the effective excitation flip angles in the MP2RAGE trains for improved accuracy of the T_1_ fit (Marques and Gruetter, 2013). An MP2RAGE image (S_MP2RAGE_) was formed based on the combination of the two complex images, S_MP2RAGE_=(S_TI1_xS_TI2_*)/(S_TI1_^2^+S_TI2_^2^), where the asterisk indicates the complex conjugate. A look-up table was used in order to estimate T_1_ maps from the S_MP2RAGE_ values (Marques et al., 2010).

### 2.4 Validation of MS-IR*EPI* using T_1_-calibrated and T_2_-calibrated ISMRM/NIST spheres

The precision and repeatability of the optimized MS-IR-EPI sequence were assessed using the International Society for Magnetic Resonance in Medicine (ISMRM)/National Institute of Standards and Technology (NIST) fiducial T_1_ and T_2_ spheres (Kenan et al., 2016). The NIST T_1_ spheres contain specific concentrations of NiCl_2_ solution to provide a known range of T_1_ values at 3T. The first ten (T_1__1 to T_1__10) calibrated T_1_-spheres, with T_1_ values ranging from 1998 ms (T_1__1) to 89 ms (T_1__10) at 3 T (Kenan et al., 2016) were used. The T_2_ spheres are doped with specific concentrations of MnCl_2_ and also provide well characterized T_1_ values at 3 T, with the first ten (T_2__1 to T_2__10) T_2_-spheres ranging from 3025 ms (T_2__1) to 293 ms (T_2__10). Note that the reference T_1_ values provided by the manufacturer for spheres T_2__1 and T_2__5 reflect measurements known to be deviant from the MnCl_2_ nominal solutions due to a problem at the time of the manufacturing process of our phantom. Due to the reduced size of the 7 T NOVA head coil compared to a 3 T head coil, home-built casings were manufactured to fit each plate of T_1_-spheres and T_2_-spheres within a separate 160 mm diameter spherical phantom that would fit within the 7 T NOVA head coil.

Due to the poor accuracy of measured B_1_ in the short T_1__7 to T_1__14 spheres (as reported in Kato et al., 2019) (see Supplementary Material, Fig. 3), only spheres T_1__1 to T_1__6, with T_1_ values within the clinically relevant dynamic range (300 to 2000 ms) of the brain tissues, were considered for analysis, in line with recent studies at 3 T (Hagiwara et al., 2019; Kato et al., 2019).

**Fig. 3:**
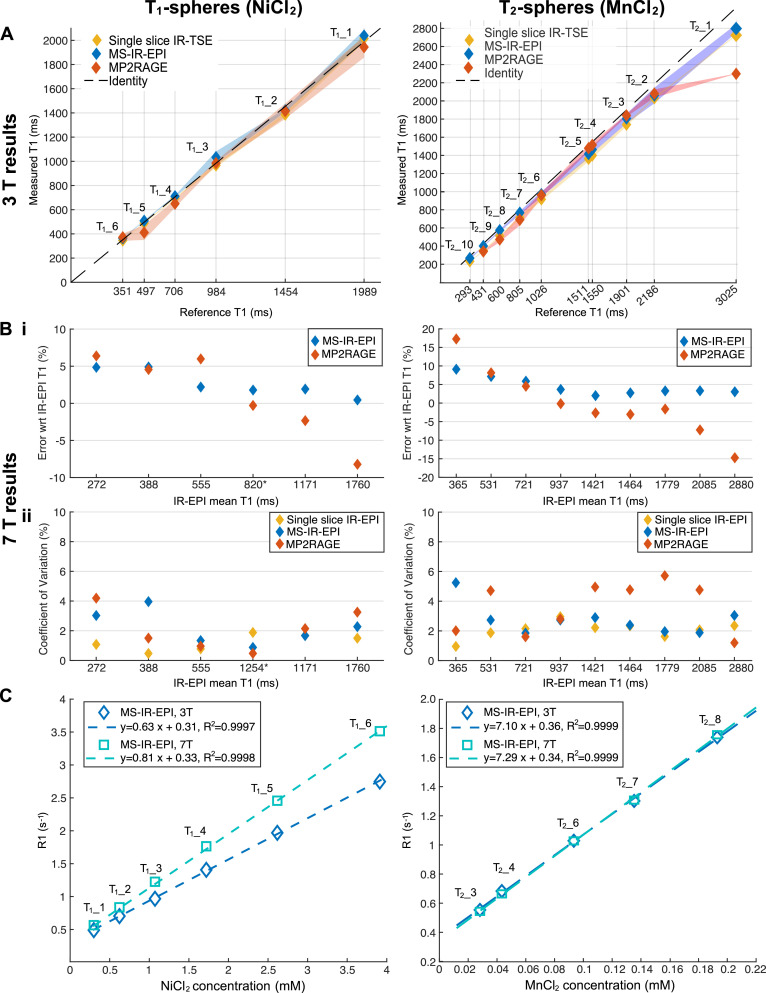
Phantom results using the ISMRM/NIST system T_1_ (left column) and T_2_ (right column) spheres. (A) Scatter plots showing linearity of measured T_1_ (ms) for the single slice IR-TSE, MS-IR-EPI and MP2RAGE plots against reference T_1_ values provided by NIST for 3 T. The error surface shows the standard deviation across 3 (T_1_-spheres) and 2 (T_2_-spheres) measurements, except for T_1__3 sphere (standard deviation across voxels from a single session). The dashed line represent identity. (B) (i) The relative deviation of MS-IR-EPI and MP2RAGE T_1_ measurements at 7 T with respect to the single slice IR-EPI T_1_ measurements. T_1_-value measures averaged across 6 (T_1__spheres) and 4 (T_2_ spheres) measurements. *Note that for T_1__3 sphere T_1_ values are average across voxels within a single measurement (see Supplementary Material). (ii) Coefficient of variation (CoV) showing the repeatability of T_1_ measures by the different methods at 7 T. *Note that the CoV for T_1__3 sphere was computed over the last 4 measurements. (D) Plots of R_1_ (s^−1^) values obtained with the MS-IR-EPI at 3 and 7 T versus the NiCl_2_ (left) and MnCl_2_ (right) nominal concentration (mM^−1^). Dashed lines represent linear regression fit. (For interpretation of the references to colour in this figure legend, the reader is referred to the web version of this article.)

To compare the accuracy of the MS-IR-EPI method with other T_1_ mapping methods, the T_1_ and T_2_ phantoms were scanned at 3T using: (i) a gold-standard IR-TSE sequence using the ISMRM/NIST recommended parameters: a single 6 mm slice with 0.98 × 0.98 mm^2^ in-plane resolution, TR/TE= 4.5 s/7 ms repeated for a range of inversion times TIs (ms) = [35, 75, 100, 125, 150, 250, 1000, 1500, 2000, 3000], 35 min acquisition, (ii) MS-IR-EPI, and (iii) an MP2RAGE acquired at 1 mm isotropic resolution (parameters in Table 1). All measurements were repeated at a second and third session for the T_1_-spheres phantom (6 and 9 months apart) and a second scan session (1 week apart) for the T_2_-spheres phantom. The temperature of each phantom (in the solution surrounding the spheres) was measured across the scan sessions. The mean T_1_ values across scan sessions was computed using each of the T_1_ mapping methods and compared to the NIST reference T_1_ values.

The phantoms were also scanned at 7 T to acquire 0.7 mm isotropic data using: (i) a single slice multi-shot IR-EPI acquisition at a range of TIs (ms) =[35, 75, 100, 150, 250, 500, 1000, 2000, 3000] with other acquisition parameters matched to the MS-IR-EPI, (ii) MS-IR-EPI (TR 5 s) and (iii) MP2RAGE sequences (parameters described in Table 1). All measurements were repeated multiple times for test and re-test reproducibility assessment. In total, 6 measurements were collected (second repeat after 40 days, and repeats 3–6 at 9 months over a 10-day period) for the T_1_-spheres phantom and 4 measurements (repeated with 2–3 day intervals) were collected for the T_2_-spheres phantom, again temperature was measured in the phantoms after each scan session. Repeatability was characterized by the coefficient of variation (CoV), defined as the ratio between the standard deviation and the mean T_1_ values across all repeat measurements (Kato et al., 2019). T_1_ values obtained with MS-IR-EPI and MP2RAGE were compared to those obtained with the reference single slice IR-EPI method.

Further scan sessions were performed in which two measurements of sequences (ii) and (iii) were collected for the NIST T_1_-spheres phantom in order to assess SNR per unit time of the generated T_1_ maps. In addition, two repeats were collected of the MS-IR-EPI sequence at a shorter TR of 3.2 s (EPI=13, TE=13 ms, 4TIs) using SMS factor 1 (92 slices) and SMS factor 2 (184 slices). To estimate image SNR, T_1_ maps generated from each individual run were subtracted to provide a ‘noise’ map. For each region of interest (ROI), SNR was estimated as the mean across voxels (in both T_1_ maps) divided by the standard deviation of the noise map. For each sequence, SNR per unit time was computed by dividing the image SNR by the total acquisition time (in minutes).

**Table 2:** Comparison of (A) T_1_ values (in ms, given by histogram peak), (B) histogram peak height and (C) full with at half maximum, for data acquired with no fat suppression (reference) and with fat suppression before and after correction using a constant *b/a* value (global correction) and (*b/a*) map (voxelwise correction). Results shown for data acquired with 70° flip angle SPIR pulse. Global correction based on *b/a* data computed from datasets of 5 and 2 FAs.

(A) T1 (ms)		Subject 1	Subject 2	Subject 3	Mean±std
FS=0° (reference)	GM	1694	1697	1706	1699±6
	WM	1135	1063	1156	1118±49
FS=70° (no correction)	GM	1372	1438	1454	1421±43
	WM	910	878	933	907±28
FS=70° corrected with (*b/a*) map	GM	1846	1831	1786	1814±31
	WM	1285	1177	1253	1238±5
FS=70° corrected with global (*b/a*)−5pts	GM	1785	1783	1814	1794±17
	WM	1194	1102	1175	1157±49
FS=70° corrected with global (*b/a*)−2pts	GM	1783	1751	1783	1772±18
	WM	1182	1090	1164	1145±49
(B) Histogram peak height with respect to reference (FS=0°)					
FS=70° (no correction)	GM	81%	81%	83%	82±1%
	WM	76%	65%	66%	69±6%
FS=70° corrected with (*b/a*) map	GM	96%	88%	107%	97±10%
	WM	103%	78%	87%	89±13%
FS=70° corrected with global (*b/a*)−5pts	GM	114%	107%	111%	111±4%
	WM	110%	85%	88%	94±14%
FS=70° corrected with global (*b/a*)−2pts	GM	114%	105%	109%	109±4%
	WM	108%	85%	87%	93±13%
(C) Histogram FWHM with respect to reference (FS=0°)					
FS=70° (no correction)	GM	137%	139%	149%	142±7%
	WM	137%	155%	173%	155±18%
FS=70° corrected with (*b/a*) map	GM	106%	117%	117%	113±6%
	WM	100%	133%	129%	121±18%
FS=70° corrected with global (*b/a*)−5pts	GM	93%	108%	108%	103±8%
	WM	93%	122%	134%	116±21%
FS=70° corrected with global (*b/a*)−2pts	GM	93%	99%	116%	103±12%
	WM	93%	122%	134%	116±21%

### 2.5 In-vivo experiments

Five subjects (2 female, age: 32±6years (mean±SEM)) were recruited to the study. Experimental procedures for all studies were approved by the University of Nottingham Medical School's Ethics Committee. All subjects gave written informed consent. None of the subjects had a history of neurological disorders.

#### 2.5.1 Effect of TR, SMS factor and number of slices on MS-IR‑*EPI* T_1_-mapping at 7 T

To assess the effect on the measured T_1_ of shortening the TR, a subject was scanned at 7 T using MS-IR-EPI protocols with TR=5 s and TR=3.2 s (MS1 and MS2 respectively in Table 1). Data were acquired using SMS=1 (96 slices) and SMS=2 (192 slices) with protocol MS1 and using SMS=1 (65 slices) and SMS=3 (195 slices) with protocol MS2, to determine whether the different RF excitation pulses used in the SMS acquisition change the measured T_1_ relative to SMS=1. In a second scan session, to assess whether T_1_ measurements are shortened due to residual MT effects from RF pulses selecting neighbouring slices, acquisitions of the MS-IR-EPI TR=3.2 s (5 TIs) protocol were repeated with varying number of slices, ns=[65, 35, 15, 5]. For this, the coverage in the slice direction across the different acquisitions was maintained by varying the slice gap: sg=[0, 0.7, 2.8, 10.5] mm for ns=[65,35,15,5] respectively. A single slice IR-EPI acquisition (with parameters matched to the MS-IR-EPI acquisition) was also collected with TI values =[320, 900, 1470, 2050, 2625] ms for comparison with the central slice of the MS-IR-EPI acquisitions. All data sets were acquired without using spectrally selective fat suppression pulses.

#### 2.5.2 Characterizing the effect of fat suppression pulses on the measured T_1_

Experiments were performed in three subjects using the 0.7 mm isotropic resolution MS-IR-EPI MS1 protocol (Table 1) to measure the longitudinal relaxation rate, R_1_ (r), for a range of nominal flip angles (*FA*) =[0°, 30°, 40°, 50°, 60°, 70°] of the SPIR (spectral pre-saturation with inversion recovery) fat suppression pulse. In addition, a dual-TR (Yarnykh, 2007) B_1_-mapping sequence (FOV=205(AP)x179(RL)x132(FH) mm^3^_,_ FA=60°, TR_1_=23 ms, TR_2_=103 ms, TE=5 s, acquisition bandwidth= 4271 (Hz), 3.2 × 3.2 × 4 mm^3^ resolution, 3 min 3 s acquisition time) was used to measure the B_1_-field distribution. A further experiment was performed in one subject where data were acquired with a set of SPIR pulses of nominal FA=[0°, 30°,45°,60°,78°,90°]. In addition, for a FA=90° two data sets were acquired, one in which the SPIR pulse was applied prior to each RF acquisition and one in which a SPIR pulse was applied prior to every alternate RF acquisition (hence using 50% of the number of FS pulses).

The measured longitudinal relaxation rate *R_1_(r)* was modelled as a weighted sum of the relaxation rates due to macromolecular relaxation and its modulation by the fat suppression pulses, which is a function of the nominal flip angle of the SPIR fat suppression pulse, *FA,* the RF field distribution*, X(r)*, and myelin density *m(r):*

(1) $$\;R_{1}\left(r\right)=a\cdot m\left(r\right)+b\cdot \mathrm{FA}\cdot X\left(r\right)\cdot m\left(r\right)$$

where *a* and *b* are constants. This model assumes that (i) the longitudinal relaxation of water protons in brain tissue is mono-exponential (in agreement with Rioux et al. (2016), which shows that the contribution from myelin water is very small and quickly decays before the shortest TI used in this study), (ii) lipid membranes, especially myelin, dominate the longitudinal relaxation and magnetization transfer of water proton spins in brain tissue, both in white matter and in cortical grey matter, and ignores the effect of iron on the longitudinal relaxivity and (iii) additional RF pulses (e.g. from fat suppression pulses) increase the longitudinal relaxation rate in water protons due to magnetization transfer from lipid membrane molecules (Kucharczyk et al., 1994).

A linear fit of *R_1_(r)* versus *FA.X(r)* was performed to estimate *α*=*a.m(r)* and *β*=*b.m(r)* from a least-squares regression, and thus evaluate the constant *(b/a)* for each voxel of the brain. This constant was then used to form a corrected R_1_ map (R_1,corr_), by removing the contribution of magnetization transfer arising from a SPIR fat suppression RF pulse with amplitude FA:

(2) $$R_{1,\mathrm{corr}}\left(r\right)=\frac{R_{1\;\left(r\right)}}{\left(1+\frac{b}{a}\cdot \mathrm{FA}\cdot X\left(r\right)\right)}$$

Corrected R_1_ maps were generated in two ways, using a voxel specific *(b/a)* parameter (voxel-wise correction) or using a constant *(b/a)* value for every voxel (global correction), defined by the peak of the histogram given by the distribution of *b/a* values across the entire brain.

#### 2.5.3 Comparison of MS-IR‑*EPI* with MP2RAGE and single slice IR‑*EPI*

Whole head images were obtained at 7 T, with 0.7 mm isotropic resolution (Table 1: protocols MS1 (SMS=2) and MP1) and at 3 T, with 1 mm isotropic resolution (Table 1: protocols MS5 (SMS=2) and MP3). These were compared with MP2RAGE T_1_ maps corrected for B_1_-inhomogeneities, and T_1_ maps obtained using a single slice IR-EPI acquisition (0.7 mm isotropic resolution, TR=5 s, EPI factor=15, TE=15 ms) repeated for multiple inversion times (TI(ms)=[305, 600, 930, 1555, 2180, 2805, 3430, 4055]) in a 4 min 50 s acquisition time.

Five subjects were scanned at 7 T. A SPIR fat suppression FA=70° pulse was applied, and a global *(b/a)* correction of the T_1_ maps performed to remove MT effects due to the FS pulses. An additional acquisition using a SPIR fat suppression FA=40° pulse was performed for Subjects 4 and 5, who did not participate in the experiment to model the impact of FS pulses (see Section 2.2.1), to compute subject-specific global *(b/a)* parameters (see Supplementary material).

In order to evaluate the SNR per unit time of T_1_ maps derived from the MS-IR-EPI and MP2RAGE sequences, the MP2RAGE and MS-IR-EPI protocols were repeated on Subject 1 at 7 T. In addition to the whole head MS-IR-EPI protocol (SMS=2, FS pulses on), additional pairs of MS-IR-EPI acquisitions were performed using SMS=1 (two repeats with and without FS pulses).

#### 2.5.4 Pushing the spatial resolution of MS-IR‑*EPI* at 7 T

To demonstrate the potential of MS-IR-EPI for high spatial resolution T_1_ mapping, a 0.5 mm isotropic resolution sequence with whole head coverage (FOV=164(RL)x192(AP)x168(FH) mm^3^) was implemented at 7 T (Protocol MS3 in Table 1) with 240 slices (SMS=3) and SPIR fat suppression FA=90°. Data were acquired with 10 slice offsets, yielding a total acquisition time of 18 min 22 s. To further highlight the potential of T_1_ mapping for cortical parcellation, the visualization of the stria of Gennari corresponding to layer IV of the primary visual cortex was compared for T_1_ maps derived using MS-IR-EPI and MP2RAGE data acquired with 0.35 × 0.35 × 0.7 mm^3^ spatial resolution collected in a coronal-oblique orientation perpendicular to the calcarine sulcus. Data were acquired at 7 T using a high density 32-channel surface receiver coil array (MR Coils, Utrecht) positioned over the visual cortex. MS-IR-EPI data (protocol MS4 in Table 1, FOV=128(RL)x80(FH)x24.5 mm^3^, SPIR fat suppression FA=60°) comprised 35 slices. Data were acquired with 5 slice offsets (5 TIs) with 3 repeats of each offset, resulting in a total scan time of 11 min 8 s. An MP2RAGE acquisition (protocol MP2 in Table1) was performed with the same slice prescription as the MS-IR-EPI (FOV=128 × 80 × 38.5 mm^3^, phase encoding in RL direction, acquisition time = 12 min 8 s). Notice these T_1_ maps were not corrected for MT effects of the fat suppression pulses, as their spatial fidelity rather than their quantitative precision was key here. Similarly, MP2RAGE data were not corrected for B_1_+ profile inhomogeneities in this comparison.

T_1_ maps for MS-IR-EPI and MP2RAGE acquisitions over the visual cortex were generated both before and after denoising, using a local complex principal component analysis (LCPCA) technique (Bazin et al., 2019). For the MS-IR-EPI, the three individual data sets acquired with the same slice offset were motion corrected using AFNI's 3dAllineate command (using mutual information as the cost function and with the centre of mass calculation restricted to within the slice plane). Motion parameters estimated using the ‘denoised’ modulus data were applied to real and imaginary original and denoised data and then averaged prior to applying T_1_-fitting algorithm.

To plot the cortical profiles of the T_1_ maps (and S_0_ maps for MS-IR-EPI), all maps were re-sampled to an in-plane resolution of 0.11 × 0.11mm^2^ using nearest neighbour interpolation; FSL's FAST algorithm was run on the re-sampled ‘de-noised’ T_1_ maps to provide WM/GM and GM/CSF boundaries which were then used to derive 20 cortical depths using the equivolume approach (Waehnert et al., 2014) implemented in the LAYNII software ( https://doi.org/10.5281/zenodo.3514297). This cortical depth segmentation was used to bin T_1_ (and S_0_) values into specific cortical depths.

## 3 3.Results

### 3.1 Optimisation of the number of acquisitions (TI values) for MS-IR‑*EPI* T_1_ mapping

The effect of the number of acquisitions used to derive the T_1_ maps using a TR of 5 s is shown in Fig. 2. A Monte Carlo simulation (Fig. 2A) showed that the accuracy of the fitted T_1_ is independent of the number of TIs used in the fit for greater than 4 TIs chosen, but that the standard deviation of the fitted T_1_ varies across slices. As the number of TIs used to derive T_1_ decreases, and with fewer permutations the minimum TI will vary significantly by slice, the variation in the standard deviation across slices increases, which can be visualized as ‘banding’ in the 3D reconstruction of the T_1_ map generated using 4 TIs but not for maps generated using 8 TIs, as shown in Fig. 2B, the effect is also less pronounced for a higher SNR level (dash lines).

### 3.2 Validation of T_1_ mapping methods on the calibrated NIST T_1_-spheres

Supplementary material Tables 1 and 2 provide the mean T_1_ values measured in the NIST T_1_ and T_2_ spheres at 3 and 7 T for each T_1_ mapping method, along with their respective NiCL_2_ and MnCl_2_ concentration and reference 3 T T_1_ values provided by ISMRM/NIST.

Fig. 3A plots T_1_ values measured at 3 T for the single slice IR-TSE, MS-IR-EPI and MP2RAGE sequences against the reference T_1_ value for the T_1_-spheres and T_2_-spheres. For the T_1_-spheres, the maximum deviation from the NIST reference T_1_ values was 4.7% (sphere T_1__2) for IR-TSE, and 5.1% (sphere T_1__3) for MS-IR-EPI, while for MP2RAGE the accuracy was 17.3% for sphere T_1__5, but 8% or better for the other spheres. The mean temperature of the T_1_-spheres phantom was 21.2 ± 0.4 °C and 20.0 ± 1.3 °C for the T_2_-spheres phantom. Linear regression analysis of the different T_1_ measures versus the reference T_1_ values showed a very strong linear correlation (1.003x - 17.2, R^2^=0.9976 for IR-TSE; 1.010x + 6.4, R^2^=0.9976 for MS-IR-EPI; 0.992x-25.6, R^2^=0.9962 for MP2RAGE) for the T_1_ spheres. For the T_2_-spheres (right), T_1_-measurements were shorter than the NIST reference T_1_ values, particularly for the MP2RAGE measurement of sphere T_2__1 (24% shorter compared to 10% and 7.6% for IR-TSE and MS-IR-EPI respectively). The accuracy was better than 9% for all spheres when using MS-IR-EPI. Since T_2__10 was below 300 ms (hence below the range of T_1_ in human brain at 3 T), this sphere was excluded from further analysis. Linear regression had a strong linear correlation (0.924x - 33.6, R^2^=0.9993 for IR-TSE; 0.9296x + 13.2, R^2^=0.9996 for MS-IR-EPI; 0.8294x+96.3, R^2^=0.9463 for MP2RAGE) for the T_2_-spheres.

At 7 T, the average single slice IR-EPI T_1_ was used as the reference to compare T_1_ values from MS-IR-EPI and MP2RAGE methods (Fig. 3B(i)). Measured T_1_ values at 7 T were shorter than at 3 T for the NiCl_2_-doped T_1_-spheres, whilst measured T_1_ values of the MnCl_2_-doped T_2_-spheres were slightly longer at 7 T (~ 3 ± 1% for the single slice IR measurement) than at 3 T. In general, MS-IR-EPI slightly overestimates T_1_ across all spheres, but accuracy is better than 5% for all spheres except the three T_2_-spheres with shortest T_1_ values. MP2RAGE T_1_ measurements in these spheres with short T_1_-values are also overestimated by more than 5%, while spheres with the longest T_1_ values (T_2__1, T_2__2 and T_1__1) are underestimated by ~14%, 7% and 8% respectively, showing poor accuracy compared with MS-IR-EPI (3.2%, 3.5% and 0.5% respectively for these spheres). The mean temperature of the T_1_-spheres phantom was 20.5 ± 1.3 °C and the T_2_-spheres phantom was 20.4 ± 0.9 °C. The repeatability of T_1_ measurements is characterized as the CoV (Fig. 3B(ii)). The CoVs of T_1_ values were smaller for the T_1_-spheres than for the T_2_-spheres, and those measured with single slice IR-EPI was lowest: < 2.8% for every T_2_-sphere except T_2__6 (3.4%), while the CoV for the T_1_ spheres was 2.2% (T_1__3) or less. For MS-IR-EPI, the highest CoV was measured for spheres T_2__9 (6.1%) and T_1__5 (4.3%), but for T_1_s> 400 ms the CoV was below 3.5%. The CoV of T_1_ values measured with MP2RAGE was the largest, larger than 5% for several T_2_-spheres (T_2__2, T_2__3, T_2__4, T_2__5, T_2__8), and the largest CoV was 3.6% (T_1__6) for the T_1_-spheres.

In order to determine the relaxivity of NiCl_2_ and MnCl_2_ at both field strengths, the longitudinal relaxation rates (R_1_) measured with each T_1_ mapping method were linearly fit versus the NiCl_2_ (for the T_1_-spheres phantom) and MnCl_2_ (for the T_2_-spheres phantom) nominal concentrations. Spheres T_2__2, T_2__9 and T_2__10 were excluded from the fit, given the poor accuracy for MP2RAGE measurements (Fig. 3B(i)), spheres T_2__1 and T_2__5 were also excluded given the unknown MnCl_2_ concentration for these spheres (see Methods). Plots for MS-IR-EPI sequence are shown in Fig. 3C (for the other sequences see supplementary material). The NiCl_2_ relaxivity at 3 T was found to be 0.66±0.01 (R^2^=0.9999), 0.63±0.01 (R^2^=0.9997) and 0.65±0.01 (R^2^=0.9529) s^−1^mM^−1^ using single slice IR-TSE, MS-IR-EPI and MP2RAGE respectively, whereas at 7 T the NiCl_2_ relaxivity was measured to be 0.86±0.01 (R^2^=0.9999), 0.81±0.01 (R^2^=0.9998) and 0.79±0.01 (R^2^=0.9997) s^−1^mM^−1^ respectively. For MnCl_2,_ similar relaxivity values were obtained at 3 T and 7 T; 8.20±0.34 (R^2^=0.9997) and 7.96±0.33 (R^2^=0.9999) s^−1^mM^−1^ for single slice IR-TSE (EPI), 7.10±0.36 (R^2^=0.9999) and 7.29±0.34 (R^2^=0.9999) s^−1^mM^−1^for MS-IR-EPI, and 9.42±0.23 (R^2^=0.9981) and 7.00±0.39 (R^2^=0.9999) s^−1^mM^−1^ for MP2RAGE.

### 3.3 Effect of TR, SMS factor and number of slices on MS-IR‑*EPI* T_1_ mapping measurements at 7 T

**Fig. 4:**
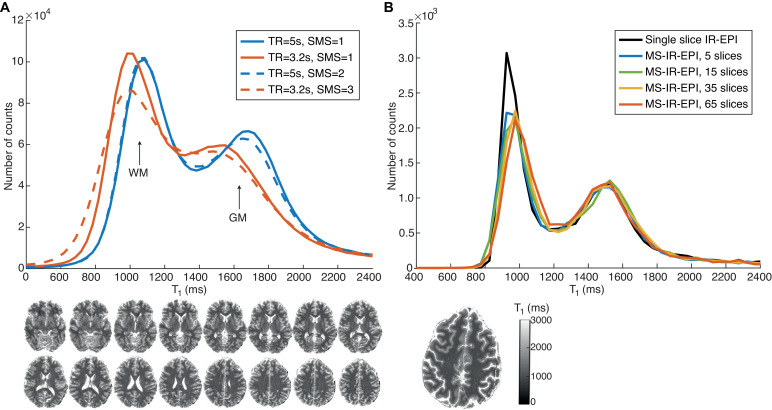
(A) T_1_ histograms comparing MS-IR-EPI data acquired with a repetition time (TR) of 5 s (blue solid line, 96 slices acquired) and 3 s (solid red line, 64 slices acquired) with simultaneous multi-slice (SMS) =1. The dash lines show corresponding T_1_ histograms acquired with SMS=2 to acquire 192 slices with TR=5 s (dash blue line) and SMS=3 to acquire 195 slices with TR=3.2 s (dash red line). All histograms correspond to the central imaging volume comprising 64 slices. T_1_ maps for every other 4 slices in this volume (acquired with TR=5 s, SMS=1) are shown at the bottom. (B) T_1_ histograms from MS-IR-EPI (TR=3.2 s, 5TIs, SMS=1) datasets acquired with a different number of slices (red: 65 slices; yellow: 35 slices; green: 15 slices and blue: 5 slices) compared to a single slice IR-EPI acquisition with matched scan parameters (TIs=[320 900 1470 2050 2625]ms). All histograms from MS-IR-EPI data correspond to the central slice, matching the single slice IR-EPI shown in the figure. (For interpretation of the references to colour in this figure legend, the reader is referred to the web version of this article.)

Fig. 4A shows the T_1_ histogram generated from data acquired with (dash lines) and without (solid lines) SMS for a TR of 5 s (blue) and 3.2 s (red). The T_1_ histograms for data acquired with SMS=1 and SMS=2 (TR=5 s) are very similar, whilst the T_1_ histogram for data acquired with SMS=3 is broader than that for the SMS=1, with a slightly lower peak for GM. Data acquired with SMS=1 shows that the reduced TR of 3.2 s (solid lines) yields shorter T_1_ values than for a TR of 5 s, particularly for GM. Fig. 4B shows that the T_1_ histograms are very similar for a central slice of the MS-IR-EPI acquisition (TR=3.2 s (SMS=1)) acquired with a different number of slices and a single slice IR-EPI data set.

### 3.4 Effect of fat suppression pulses on T_1_ measurements

**Fig. 5:**
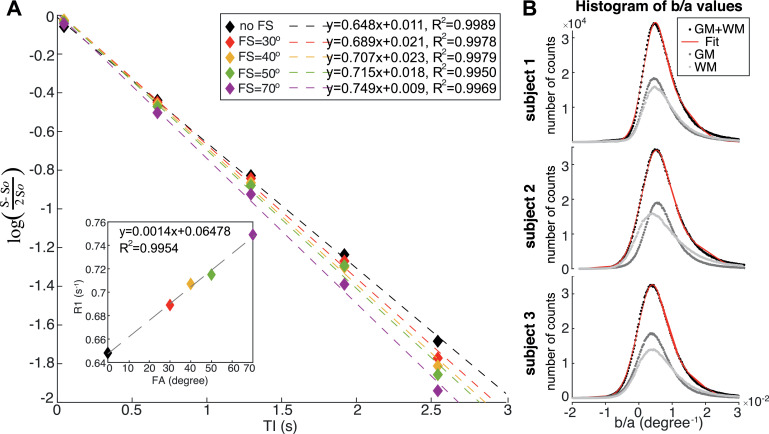
(A) Logarithmic plot of the signal intensity within a region of interest in the corpus callosum (single slice, subject 2) versus inversion time for data acquired measured for a MS-IR-EPI dataset with no fat suppression (black diamonds) and with different fat suppression (FS) SPIR pulse flip angle (coloured diamonds). The logarithmic y-axis plots the difference in signal intensity (S) at each of the first 5 inversion times compared to the last inversion time (S_0_). The dash lines plot the linear fit for each data set, showing strong linearity (R^2^ >0.99). Each fit's gradient corresponds to the longitudinal relaxation rate (R_1_) which increases with increasing fat suppression power. The inset shows that the fitted R_1_ is linearly correlated with the SPIR pulse flip angle. (B) Histogram of *b/a* parameter values obtained by linear regression of R_1_ versus SPIR FS flip angle for GM tissue (dark grey), WM tissue (light grey) and total (GM+WM) tissue (black) for each subject. Red line: double Gaussian fit to the (GM+WM) data. (For interpretation of the references to colour in this figure legend, the reader is referred to the web version of this article.)

The model of the effect of fat suppression on longitudinal relaxation rate (Eqn.(1)) assumes the longitudinal relaxation of water protons in brain tissue is mono-exponential, whether or not there is further relaxation due to magnetization transfer. Logarithmic plots of the MS-IR-EPI signal intensity (within a ROI in the corpus callosum) versus inversion time indeed show a very strong linear relation (R^2^>0.99) for each of the data sets acquired with different fat suppression levels (Fig. 5A). There is also a strong linear correlation of the fitted R_1_ values versus fat suppression SPIR flip angle (R^2^=0.9954) (Fig. 5A inset). Fig. 5B shows a histogram of the *b/a* parameter obtained by voxel-wise linear regression of R_1_ versus SPIR FS flip angle for GM (dark grey), WM (light grey) and total (WM+GM) tissue (black) for the three subjects scanned. The distribution of *b/a* is very similar for GM and WM, hence the distribution of total GM and WM tissue values across the brain was fit to a two Gaussian mixture model (red line), and the mode of highest amplitude Gaussian used as a constant parameter for the global correction method; this was found to be very similar across subjects (with a mean value of 0.0045±0.0002°^−1^). The voxel-wise correction introduces propagated noise in the corrected R_1_ maps. Given that the measured voxel-wise *b/a* correction factor shows a very slow spatial variation compared with structural details, we were able to improve the SNR of the corrected R_1_ maps when using the voxel-wise correction by spatially smoothing the *b/a* parameter maps with a 2 mm Gaussian kernel. Larger kernels did not further reduce the standard deviation in the corrected R_1_ maps.

**Fig. 6:**
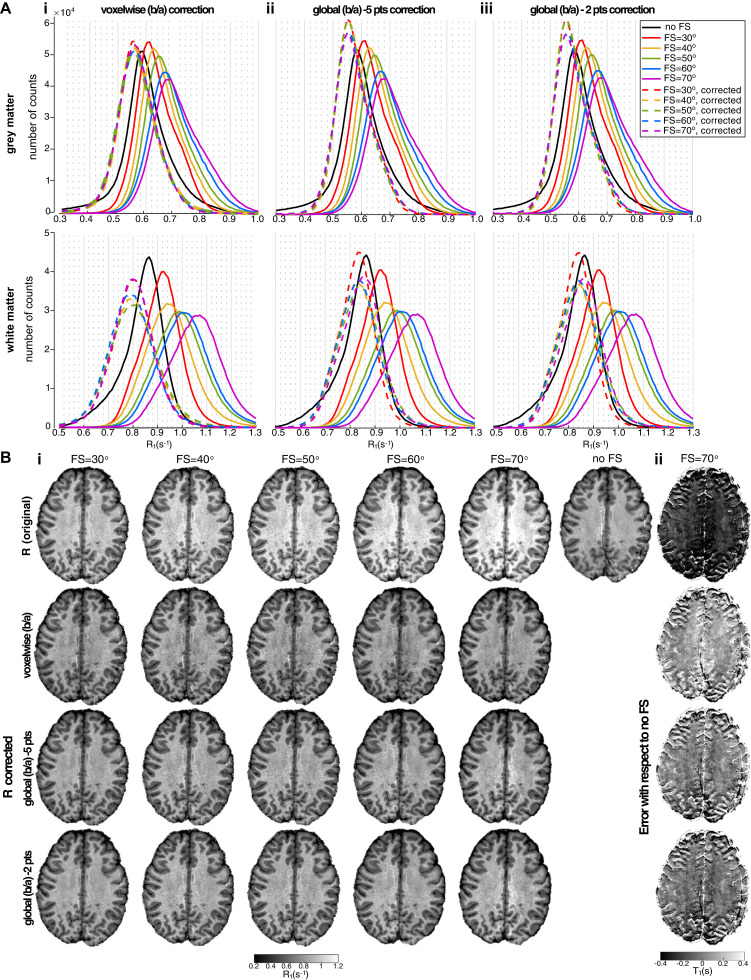
(Ai) R_1_ histograms in grey matter (top) and white matter (bottom) for one subject's data acquired with different levels of fat suppression (FS) by varying the flip angle of the SPIR pulse. The black line represents data acquired with no FS and the colour solid lines represent data acquired with FS SPIR flip angles ranging from 30° (red) to 70° (violet). The dashed colour lines show corresponding R_1_ histograms after correction using: *b/a* data generated by linear regression of R_1_ versus SPIR flip angle to correct (Eqn. (2)) using: (i) voxelwise correction (the *b/a* parameter map was spatially smoothed by a Gaussian kernel with a full width at half maximum (FWHM) of 2 mm prior to correction) and a global *b/a* correction with the constant *b/a* parameter value computed from (ii) the full dataset comprising 5 FS flip angles of 30°, 40°, 50°, 60° and 70° and (iii) a reduced dataset of two FS flip angles of 40° and 70° only. (B) (i) Original R_1_ maps (first row) shown for the different SPIR flip angles (including data acquired with no fat suppression, right) and corresponding R_1_ maps after correction with the different methods. (ii) Difference image between T_1_ maps for data acquired with SPIR FA=70° (uncorrected and corrected with the different methods) and data acquired with no FS. (For interpretation of the references to colour in this figure legend, the reader is referred to the web version of this article.)

The default RF power for the ‘strong’ SPIR fat suppression pulse in the Philips system corresponds to a FA of 120°, with a FA of 90° assigned to ‘medium’ and 60° to ‘weak’ power respectively. For the whole-head MS1 protocol (Table 1), these pulse strengths result in SAR levels of 1.5, 2 and 2.7 W/Kg respectively. We observed that a SPIR fat suppression pulse with FA of 70° was sufficient to effectively suppress the fat artefact with 19.2 FS pulses per second (Fig. 6). This resulted in a 41% reduction in the SAR level of the sequence compared to the default ‘strong’ FS power. Fig. 6A shows that the position of the peak of the R_1_ histogram increases as the SPIR fat suppression flip angle increases (solid colour lines) as compared to no fat suppression (black line). Table 3A summarises the T_1_ values at the histogram peaks in GM and WM for data acquired with no fat suppression and with 70° flip angle SPIR pulses. When we used 70° flip angle SPIR pulses, GM T_1_ values were shortened by 17% (Subject 3) to 23% (Subject 1) and WM T_1_ values by 21% (Subject 2) to 25% (Subjects 1), compared with no fat suppression. R_1_ maps collected across SPIR flip angles were linearly fit to Eq. (1) to generate a *b/a* parameter map. Histograms of R_1_ maps corrected (Eq. (2)) using the voxel-wise method are shown in Fig. 6Ai, whilst Fig. 6Aii (and 6Aiii) show R_1_ histograms corrected using a constant *b/a* parameter value (global correction). The R_1_-corrected histograms (dash lines) are similar across fat suppression levels and match the data acquired with no fat suppression (black line), with a narrower distribution (smaller FWHM and higher peak) than the uncorrected data, particularly for the global correction method (see Table 3 for measures for the data acquired with FS flip angle of 70°). The histogram peaks for the R_1_-corrected data are closer to the reference (no fat suppression data), with slightly longer T_1_ values 7.1 and 5.6% of (GM) and 10.7 and 3.5% (WM) (across subjects) for voxelwise and global correction respectively. The use of a reduced dataset of two R_1_ maps with SPIR FAs of 40° and 70° to derive the global *b/a* correction parameter yields results (Fig. 6Aiii) very similar to the full data set (Fig. 6Aii). The maximum difference in corrected-R_1_ (compared to correction using the full data set) was 1.8% for GM and 1.1% for WM in Subject 2 (Table 3).

**Table 3:** T_1_ values of histogram peak for data acquired with standard single slice IR-EPI, MS-IR-EPI with fat suppression (both same slice as the standard IR-EPI and whole brain) and whole brain MP2RAGE.

T_1_ (ms)		Subject 1	Subject 2	Subject 3	Subject 4	Subject 5	Mean±std
Single slice	GM	1790	1730	1688	1762	1758	1746±39
IR-EPI	WM	1010	1090	1083	1108	1092	1077±38
MS-IR-EPI	GM	1766	1790	1798	1770	1828	1790±25
(single slice)	WM	1077	1170	1176	1158	1168	1151±42
MS-IR-EPI (whole brain)	GM	1690	1766	1806	1845	1808	1783±60
	WM	1110	1155	1200	1195	1192	1170±38
MP2RAGE (whole brain)	GM	1641	1615	1635	1685	1688	1653±32
	WM	1100	1135	1145	1155	1165	1140±25

Fig. 6Bi shows the similarity of the voxel-wise *b/a* R_1_-corrected maps (second row) with R_1_ map after correction with a global constant *b/a* (third and fourth rows for regression using data with 5 and 2 FS flip angles respectively). The corrected R_1_ maps are generally similar to those acquired with no fat suppression (no-FS, Fig. 6Bii). The global correction shows smaller variance than the voxel-wise correction, appearing similar to the no-FS map but with the fat artefact removed. Table 3A shows that the T_1_ values obtained with both correction methods are similar, although slightly longer for the voxel-wise *b/a* correction method, particularly for WM (maximum difference of 3.4% for GM and 7.7% for WM in Subject 1). The R_1_ histograms for the global correction display a higher peak and narrower FWHM, reflecting the increased SNR compared to the voxel-wise method. It should be noted, however, that when a B_1_-map is unavailable the voxel-wise method can be used to remove spatial variations due to both an inhomogeneous RF transmit field and attenuation of the water signal by the spectrally selective FS pulses in areas of inhomogeneous B_0_ (see Supplementary Material, Fig. 4). Effective correction can also be achieved by collecting two data sets with FS flip angle (90°), but where data are collected with an FS pulse prior to each acquisition in one map and prior to alternate acquisitions in the other map, such that MT effects are exactly halved.

### 3.5 Comparison of MS-IR‑*EPI* with MP2RAGE and single slice IR‑*EPI* T_1_ mapping

**Fig. 7:**
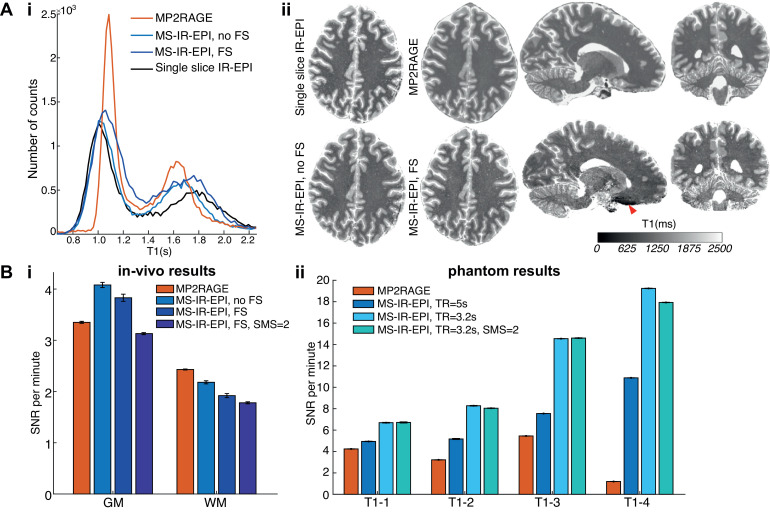
(A) Comparison of T_1_ maps obtained with the different schemes at 7T. (i) T_1_ histograms for a single slice (shown in ii) for single slice IR-EPI (black line), MP2RAGE (orange) and MS-IR-EPI with (dark blue) and without (light blue) fat suppression. Notice that the data acquired with fat suppression had been corrected as described in the methods. (ii) Comparison of whole brain T_1_ maps (sagittal, coronal and axial views) obtained with MP2RAGE (top raw) and MS-IR-EPI acquired with fat suppression pulses and SMS=2 (bottom raw). Axial view also shown for T_1_ map generated with MS-IR-EPI and single slice IR-EPI schemes without fat suppression. (B) SNR per unit time (minute) comparison in (i) GM and WM tissue ROIs for T_1_ maps generated with MP2RAGE (orange) and MS-IR-EPI schemes without fat suppression (using SMS=1) pulses and with fat suppression pulses for SMS=1 and SMS=2. (ii) NIST sphere for T_1_ maps generated with MP2RAGE (orange) and MS-IR-EPI (different shaded of blue). Errorbars represent the standard deviation across two repeat measurements. (For interpretation of the references to colour in this figure legend, the reader is referred to the web version of this article.)

T_1_ histograms of a single slice are shown in Fig. 7Ai with distinct WM and GM peaks. Compared to a multi-shot single slice IR-EPI (black line), the GM peak is similar for no FS (light blue) and after correction (dark blue), but the WM peak T_1_ is longer for the corrected R_1_ sequence (see Table 3: across subjects the T_1_ values for MS-IR-EPI are longer than a single slice IR-EPI by 1.6% and 6.3% in GM and WM respectively). MP2RAGE gives a WM peak T_1_ similar to MS-IR-EPI (with FS) whereas the GM peak is shorter. This pattern is repeated for the whole brain volume (Table 3), with mean WM T_1_ of 1170±38 ms and 1140±25 ms measured with MS-IR-EPI and MP2RAGE sequences respectively (*n* = 5), and mean GM T_1_ of 1783±60 ms for MS-IR-EPI compared to 1653±32 ms for MP2RAGE. Fig. 7Aii compares T_1_ maps generated with MS-IR-EPI and MP2RAGE in axial, sagittal and coronal views. The red arrow indicates the effect of the large B_0_ field gradient in regions near air-tissue interface, causing larger distortions in MS-IR-EPI than MP2RAGE.

The SNR-per-unit-time for MS-IR-EPI and MP2RAGE measures of T_1_ at 7 T in the NIST/ISMRM spheres and *in-vivo* are shown in Fig. 7Bii. Data for the phantom shows the SNR-per-minute is higher for MS-IR-EPI (blue) than MP2RAGE (red) in all spheres, particularly for the TR=3.2 s protocol. Also note that the SNR per unit time is only slightly decreased when using SMS=2 compared to SMS=1. In contrast, SNR-per-minute in the brain (Fig. 7Bi) is larger for T_1_ in GM tissue measured with MS-IR-EPI for SMS=1 but is reduced below the level of MP2RAGE when using SMS=2 with FS and for all MS-IR-EPI measures in WM.

### 3.6 Pushing the spatial resolution of MS-IR‑*EPI* at 7 T

**Fig. 8:**
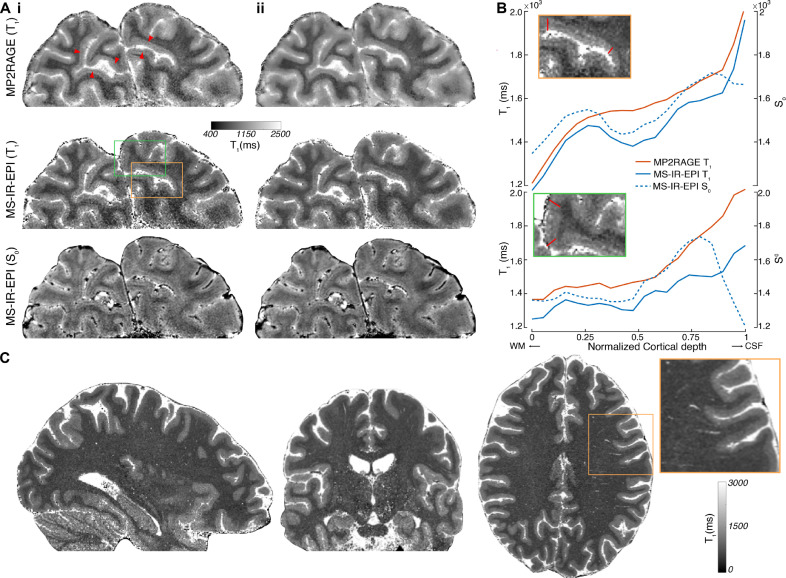
(Ai) High spatial resolution (0.35 × 0.35 × 0.7 mm^3^) T_1_ maps generated from MP2RAGE data (T1_1_/T1_2_=900/2375 ms, TR_shot_=4.5 s, 55 slices, 12 min 8 s acquisition time; top raw) and fitted T_1_ (middle raw) and S_0_ (bottom raw) maps derived from MS-IR-EPI data (TR=3.2 s, TE=20 ms, EPI factor=13, 35 slices, offsets=[0,7,14,21,28], 3 averages, 11 min 8 s total acquisition time) for an example slice of a partial brain dataset. The red arrowheads point to the Stria of Gennari. (ii) Corresponding maps after complex de-noising. (B) Cortical profiles for each of the original data sets (without de-noising) shown in (A) – mean across profiles between the two boundaries indicated by the red lines in the insets (top left) for a region within the striate cortex (top) and a region outside the striate cortex (bottom). Cortical profiles are plotted from the WM boundary to CSF boundary. Profiles from the striate cortex show a dip in T_1_ (and S_0_), corresponding to the Stria of Gennari, for the MS-IR-EPI data (solid and dashed blue profiles, respectively) but not for the MP2RAGE data (orange), whereas profiles outside the Stria do not show a dip. (C) Whole head MS-IR-EPI T_1_-data acquired at 0.5 mm isotropic resolution (EPI factor=7, TE=14 ms, TR=3.8 s, offsets=[0,8,16, 24, 32, 40, 48, 56, 64, 72], SMS=3, 240 slices, 18 min 44 s acquisition time). (For interpretation of the references to colour in this figure legend, the reader is referred to the web version of this article.)

High spatial resolution (0.35 × 0.35 × 0.7 mm^3^) T_1_ maps of the visual cortex generated with MS-IR-EPI and MP2RAGE are shown in Fig. 8A. The arrows indicate the presence of a hypointense band within the calcarine sulcus corresponding to the Stria of Gennari. This can be observed in T_1_ maps generated with MP2RAGE (top row) and MS-IR-EPI (middle row), as well as in the S_0_ parameter fit for MS-IR-EPI (bottom row). The visual appearance of the MP2RAGE data is in line with previous work (see Waehnert et al., 2016) in which the Stria of Gennari is typically only sporadically visible, this is also the case for the MS-IR-EPI data (Fig. 8A(i)). Intracortical profiles from WM to the CSF boundary are plotted for striate and extra-striate cortex (for the original data before de-noising) in Fig. 8B, showing a clear mid-cortical dip in T_1_ and S_0_ values for the MS-IR-EPI data within striate cortex compared to extra-striate cortex, but not in T_1_ values from the MP2RAGE data. The S_0_ image from the MS-IR-EPI data is also of notably very high quality, and may aid the definition of intracortical veins. Due to the MS-IR-EPI data exhibiting reduced blurring, after complex denoising (Fig. 8A(ii)) the visualization of the Stria of Gennari is improved for MS-IR-EPI data but not for MP2RAGE data. Fig. 8C shows whole head 0.5 mm isotropic T_1_ maps generated with MS-IR-EPI in under 19 min (note these data have not been de-noised).

## 4 Discussion

We have implemented a fast multi-slice multi-shot inversion-recovery 2D-EPI T_1_ mapping method, which completely fills the time available after each inversion pulse with acquisition of k-space segments of each slice, minimizing any dead-time and limits geometric distortions and image blurring. We combined this sequence with slice offset sampling and simultaneous multi-slice imaging to generate time-efficient T_1_ maps of the whole brain with sub-millimetre spatial resolution. Monte Carlo simulations allowed the minimum number of acquisitions needed to obtain consistent T_1_ values across slices to be determined. Banding in the slice direction may originate from phase errors in regions where the longitudinal magnetization is near zero. Simulations show that as the number of TIs and SNR increases the ‘banding artefact’ becomes less prominent (Fig. 2A(ii)). Cohen and Polimeni (2017) have shown that the slice ordering can also be optimized to reduce the required number of measurements without adversely affecting the quantitative accuracy and precision of the T_1_ maps.

The MS-IR-EPI results were compared with those using a single slice IR-EPI sequence and the widely used 3D MP2RAGE T_1_ mapping with B_1_-correction (Marques et al., 2010; Van de Moortele et al., 2009; Haast et al., 2018; Marques and Gruetter, 2013). The MS1 protocol with TR=5 s is optimal to accurately measure T_1_ in the brain at 7 T, whereas for applications where the precision of the T_1_ value is not critical, such as cortical depth analysis (Huber et al., 2016; Kashyap et al., 2018) or cortical parcellation where relative changes are important, protocols with shorter TR may be preferred to speed up the acquisition.

### 4.1 Validation of T_1_ mapping methods on the calibrated NIST T_1_-spheres

T_1_ values obtained with MS-IR-EPI at 3 T were consistent with the reference T_1_ values of the T_1_-spheres (R^2^=0.9976) and T_2__-_spheres (R^2^=0.9767) provided by NIST. We report, for the first time, quantitative T_1_ values of the ISMRM/NIST T_1_-sphere system and T_2_-sphere system at 7 T. At 7 T, both the T_1_ mapping accuracy (with respect to the single slice IR measure) and repeatability of MS-IR-EPI were superior to that of MP2RAGE. In particular, for long T_1_ values (>1800 ms) MS-IR-EPI provides good accuracy while MP2RAGE underestimates T_1,_ suggesting that longer TR_shot_ is required for MP2RAGE to accurately measure T_1_ in grey matter tissue. The accuracy of both MS-IR-EPI and MP2RAGE decreases for low T_1_ values (<500 ms), which are below the physiological range of T_1_ in the human brain at 7 T.

It is worth noting that although we show a reduction in the sphere T_1_ values at 7 T as compared with 3 T, which at first may seem inconsistent with the commonly expected increase in T_1_ with field strength (Yen et al., 2017), we have found that this is a predictable result of the increase of the relaxivity (*r*) of NiCl_2_ between 3 T (~0.63 s^−1^mM^−1^) and 7 T (~ 0.81 s^−1^mM^−1^) measured with MS-IR-EPI. This is consistent with previous work showing a relaxivity of NiCl_2_ at 7 T of 0.93±0.23 s^−1^mM^−1^ (Rooney et al., 2007) compared to lower measures at lower field strengths (ranging from 0.2 to 4T). Our measure of relaxivity at 3 T is also in agreement with previous work reporting an *r* of 0.620 s^−1^mM^−1^ (Thangavel et al., 2017). We found that our measures of relaxivity of MnCl_2_ were similar at 3 and 7 T (7.10±0.36 and 7.29±0.34 s^−1^mM^−1^ respectively for MS-IR-EPI) and comparable to *r* values for MnCl_2_ reported in the literature: 6.397 s^−1^mM^−1^ (Thangavel et al., 2017) and 7.4 s^−1^mM^−1^ (Nofiele and Cheng, 2013) at 3 T, and 6.37 s^−1^mM^−1^ (Castets et al., 2015) at 7 T. Although the accuracy (with respect NIST T_1_ reference values) was better for the T_1_-spheres than the T_2_-spheres, the T_2_-spheres are better suited for 7 T imaging as they offer a wider range of T_1_-values which more closely match those found within the human brain compared to the T_1_-spheres, due to the increased relaxivity of NiCl_2_ at 7 T.

### 4.2 MT effects on measured T_1_ values due to spectrally selective fat suppression pulses

MS-IR-EPI data were collected using a range of SPIR fat suppression flip angles. Our results show that spectrally selective fat suppression pulses decrease the apparent value of T_1_, with a severe underestimation when using high power FS pulses (Fig. 6). It has been previously demonstrated using multi-slice Look-Locker imaging (Shin et al., 2009) that frequency-selective fat suppression pulses can invoke magnetization transfer contrast through the exchange between free and motion-restricted protons. Cohen and Polimeni (2018) investigated the effects of the fat suppression pulses in T_1_ measurements of the NIST calibrated T_1_-spheres phantom and showed that the T_1_ shortening effect of the fat suppression pulse was particularly pronounced for phantom compartments with long T_1_. Although the study by Polders et al. (2012) using single-shot MS-IR-EPI with SPIR fat suppression pulses at 7 T found that fat suppression pulses did not significantly affect the estimated T_1_ values in a human brain, Renvall et al. (2016) showed maps at 3 T with reduced T_1_ when fat suppression was applied. This is in line with a more recent study (Kashyap et al., 2018) reporting T_1_ values at 7 T using single shot multi-slice EPI with fat suppression pulses (GM:1460±250 ms, WM:970±150 ms:comparable to the T_1_ values observed in our study with SPIR FA=70°) which were considerably shorter than those measured with MP2RAGE (GM:1666±220 ms, WM:1180±90 ms). Note that the degree of reduction in T_1_ values may vary across MR vendors as the fat suppression implementation is not identical across vendors. This discrepancy above can be explained by the small number of fat suppression pulses used (4.6 pulses per second) by Polders et al. compared to the Kashyap et al. study (11.7 pulses per second) and the multi-shot MS-IR-EPI sequences used in the present study (19.2 pulses per second for MS1, Table 1). We indeed observed that halving the number of fat suppression pulses in the sequence had a dramatic effect in the measured T_1_ values (for high power SPIR pulses, Supplementary Material Figure 4). This is consistent with Shin et al. (2009) who showed that the fractional loss by magnetization transfer effects was more severe for shorter repetition times and a greater slice number.

Shin et al. (2009) suggested the use of water-selective binomial composite pulses to suppress the fat artefact while minimizing MT effects. However, these water-selective binomial pulses are effective only for low flip angles and large slice thicknesses, conditions which are ideal for a Look-Locker sampling scheme and 3D readouts where low flip angles are used, but not useful for high spatial resolution 2D-EPI acquisitions. We also investigated whether magnetization transfer effects due to off-resonance RF irradiation from the slice selective pulses of neighbouring slices had any significant effect on the T_1_ value (Santyr, 1993; Wright et al., 2008). Comparison of T_1_ maps generated with varying number of slices, in the absence of fat suppression pulses showed that this was not the case (Fig. 4).

To correct R_1_ measures for MT effects of the fat suppression pulses, we modelled the measured longitudinal relaxation rate as a function of (i) the myelin density, (ii) nominal flip angle of the spectrally selective FS pulses and (iii) the RF field distribution. Our model assumes that the R_1_ of water protons in brain tissue is mono-exponential. Rioux et al. (2016) show this to be valid for TI values much greater than that of the short T_1_ component, associated with myelin water (Labadie et al., 2014) of the order of 57 ms. Our MS-IR-EPI sequence does not include inversion times short enough to sample this short T_1_ component, and the logarithmic plots of the MS-IR-EPI signal intensity versus inversion time show a very closely linear relation, for a wide range of nominal FS pulse flip angles (Fig. 5A). Note that the mono-exponential assumption fails at the boundary between tissues, but partial volume effects are alleviated by increasing spatial resolution,

Our correction of R_1_ values for MT effects is based on the ratio of *b/a* values (Eqn. (1)), which was found to be strikingly similar for GM and WM tissue in all three subjects scanned, in agreement with the linear relaxometry model of Callaghan et al. (2015). We compared two methods of correcting the R_1_ maps for MT contribution, a voxel-wise correction using the *b/a* parameter map or a global correction using a constant value of *b/a* from the peak of the (*b/a*) histogram. Both methods worked well (Fig. 6) to correct undesired MT effects in the T_1_ quantification and yielded very similar T_1_ values for GM (1.1% longer for voxel-wise correction) although T_1_ values were slightly longer in WM (6.5%) using the voxel-wise correction (Table 3). Our T_1_ values for GM (1794/1814 ms for global and voxel-wise correction) are close to those reported in the literature (Wright et al., 2008; Polders et al., 2012; Dieringer et al., 2014) and to those obtained with single slice IR-EPI (Table 3). Shorter T_1_ values (5.6% and 6.8% for global and voxel-wise correction respectively, Table 3) were obtained with the MS-IR-EPI acquisition with no fat suppression. The subject-averaged WM corrected T_1_ values (1157±49 and 1232±68 for global and voxel-wise correction respectively) are in line with values reported in the literature, ranging from 1074/1120 ms measured using MS-IR-EPI (Polders et al., 2012), 1220 ms using MP2RAGE (Metere et al., 2017; Caan et al., 2019) and 1284 ms using IR-SE (Dieringer et al., 2014), but shorter than the long T_1_ component (1349 ms) measured with IR-SE (Rioux et al., 2016). Direct comparison of T_1_ values measured with MS-IR-EPI and reference IR-EPI in a single slice (for two subjects) largely agree, with very slightly longer T_1_ (2.5% and 6.9% in GM and WM respectively) measured with MS-IR-EPI. Thus, the proposed correction method appears to enable accurate T_1_ quantification with MS-IR-EPI when using spectrally selective fat suppression pulses.

#### 4.2.1 Global versus voxelwise FS correction

The global *b/a* FS correction method produces artefact-free maps, as expected, regardless of the power of the FS pulses used in the *b/a* parameter estimation. Since a SPIR FS pulse with FA=70° is required to completely remove the fat ring image artefact (Fig. 6B), this artefact appears in the correction parameter maps when lower FS pulse values are used in their generation, and hence also in the corresponding T_1_ maps when the voxel-wise correction is used. In order to achieve voxel-wise-corrected images free from fat artefact, at least two acquisitions are required, one using the minimum FA for the FS pulse to effectively remove the fat artefact, and a second with a higher FA where the MT effects are very strong (see Supplementary Material, Fig. 4). We found that when only two (40° and 70°) of the five FS flip angle datasets are used, very similar mean values for R_1_ are obtained (1.2% lower for GM, and 1% lower in WM), indicating that the accuracy of the global correction method is not compromised when fewer datasets are used. This is in contrast with the voxel-wise correction, where fewer data sets results in a wider histogram of R_1_ values (see Supplementary Material, Fig. 4), suggesting undesired noise propagation from the fitted *b/a* values into the voxel-wise corrected maps. However, the voxel-wise method can remove spatial variations due to inhomogeneous RF transmit field without requiring the acquisition of a separate B_1_-map and can correct for signal attenuating caused by the spectrally selective FS pulses in regions with poor B_0_ (see Supplementary Material, Fig. 4).

We recommend the global *b/a* correction method as this provides accurate T_1_ values while introducing minimal noise into the T_1_ maps. This method carries the implicit assumption that the relationship between the longitudinal relaxation described by intrinsic T_1_ and the magnetization transfer rate remains constant across healthy brain voxels, which would hold if both T_1_ and MT relaxation are indeed dominated by the membrane lipid content of each voxel (essentially the myelin content), rather than by iron or brain proteins for example. This assumption is supported by recent work (Leuze et al., 2017) showing that T_1_ and MT become uniform in brain tissue cleared of lipids. Our observation that the mode of the *b/a* histograms of grey and white matter are similar in magnitude (Fig. 5B) also supports this assumption.

Given the stability across subjects of the *b/a* correction parameter value, the mean value across a group of subjects could be hence used as a MT correction parameter for all acquisitions, once this parameter value has been determined for the desired version of the MS-IR-EPI sequence on a specific MRI scanner. It is important to note however that the age dependency was not explored in this study, and it might be possible that during brain development or ageing the *b/a* correction parameter will fluctuate. Hence, when studying different groups of participants, a subject-mean global *b/a* value characteristic of the group under consideration may be needed.

#### 4.2.2 Accuracy of T_1_ mapping using mono-exponential models

There has been much discussion of the accuracy of T_1_ measurements. T_1_ mapping experiments are typically analysed using a mono-exponential model, thereby assigning a single longitudinal relaxation time constant to the magnetization within the voxel. Accuracy can of course be affected by the choice of optimal sequence parameters (Weiss et al., 1980), the fitting model (Li et al., 2010) and by slice profile imperfections. It has been suggested, however, that the assumption of mono-exponential signal recovery is itself incorrect. Some authors claim that magnetization transfer between free water protons and macromolecule-bound protons itself introduces a second exponential component (Rioux et al., 2016; Rui et al., 2020) giving rise to the divergence of published T_1_ values. The bi-exponential recovery of longitudinal magnetization observed in most tissue types, including WM and GM tissue (Gochberg and Gore, 2007; Prantner et al., 2008) has been attributed to this effect, although the short and long T_1_ components are hard to relate to the presumed free water and bound proton pools. The single pool model has been shown not to be fully valid at 3 and 7 T for the relaxation of proton spins in white matter. Work by Labadie et al. (2014) showed unequivocally a short-T_1_ contribution from myelin water, trapped between the myelin layers wrapping myelinated axons. Later work (Rioux et al., 2016) using IR-TSE imaging to characterize the long and short components of T_1_ relaxation in WM agreed with this study, and measured a short component of T_1_=48 ms (9%) and T_1_=57 ms (13%) at 3 and 7 T respectively. They showed, however, that for sequences that acquire data using multiple inversion times, as performed for the proposed MS-IR-EPI sequence, a mono-exponential model is precise for measuring the long T_1_ component.

Interestingly, Rioux et al. (2016) also evaluated the effect of the short T_1_ component on T_1_ estimation using MP2RAGE, since the signal equations used to convert the intensity of a composite MP2RAGE image into a T_1_ value assume a mono-exponential T_1_ recovery. They found that the effect of the short T_1_ component cannot be mitigated as easily as in sequences which use multiple IRs, with significant differences (90 and 125 ms at 3 and 7 T respectively) with respect to the T_1_ long component.

### 4.3 Comparison of whole brain measures between MS-IR‑*EPI* and MP2RAGE

In comparison with MP2RAGE, T_1_ values measured with MS-IR-EPI were similar (2.6% longer) in WM but longer (7.9%) in GM. However, our MP2RAGE T_1_ measure for GM (1653±32 ms, average across 5 subjects) is shorter than reported in other studies using MP2RAGE, where values of 1800 ms (Metere et al., 2017), 1959 ms (Caan et al., 2019) and 1870–1979 ms (Marques et al., 2010) are stated. Only one of these studies (Caan et al., 2019) used the same scanner vendor, but the authors also used a longer TR_shot_ (6 s) than the 5 s used in the present study, which could potentially result in longer T_1_ estimates. In our study, the 5 s time between inversion pulses was chosen to match the TR for the MS-IR-EPI sequence. A recent study using the same TR_shot_=5 s for MP2RAGE reported a very similar T_1_ value (1660±35 ms) for GM tissue to results reported in this manuscript. These observations, taken together with our phantom results suggest that the MP2RAGE sequence underestimates T_1_ values > 1800 ms, as a TR_shot_ of 5 s is too short to accurately measure GM. In contrast, for the MS-IR-EPI sequence, T_1_ could be accurately measured in GM (yielding comparable measures to single slice IR-EPI). It should be noted that the longitudinal magnetization has more time to freely recover in MS-IR-EPI compared with MP2RAGE, where the longitudinal magnetization is continuously sampled over the long GRE trains.

Both MS-IR-EPI and MP2RAGE sequences use adiabatic inversion pulses, which makes these sequences less sensitive to B_1_-field inhomogeneities than other sequences, such as the Variable Flip Angle method (Stikov et al., 2015). Simulations for different levels of the adiabatic inversion on the accuracy of T_1_ measured show that for target T_1_ value of 2000 ms, the error is only 10.7 ms (0.5%) when the efficiency is 90%, increasing to ~30 ms (1.5%) when efficiency is further reduced to 70%, and this error decreases for shorter T_1_ targets (see Supplementary Material, Fig. 2). For the MP2RAGE sequence, B_1_-transmit field inhomogeneities cause deviations from the nominal flip angles in the GRE trains, since the longitudinal magnetization is continuedly sampled during the GRE acquisition train, errors in the FA will affect the accuracy of T_1_ estimates. In contrast, for MS-IR-EPI the longitudinal magnetization experiences a single excitation pulse. Deviations from the nominal excitation FA means that the longitudinal magnetization will not be fully saturated, which will have an impact in the SNR. For MS-IR-EPI, the largest impact on T_1_ accuracy due to the varying B_1_ transmit field is due to the spectrally selective fat suppression pulse, given that there is a strong dependence of T_1_ with the fat suppression FA. Hence, when using spectrally selective fat suppression pulses, knowledge of the B_1_-field distribution is crucial when correcting MT effects from the T_1_ maps.

The quality of the whole-brain T_1_ maps for MS-IR-EPI is similar to that of MP2RAGE (Fig. 7). The MP2RAGE acquisition takes longer, but provides a higher SNR compared to the faster MS-IR-EPI T_1_ map, particularly in WM, as evidenced by the sharper histogram peak for MP2RAGE. Multi-shot EPI acquisitions are more robust against B_0_ inhomogeneity than single shot EPI because the lower EPI factor entails a short acquisition window, minimizing geometric image distortions and dropouts. However, the large B_0_ field gradient in regions near air-tissue interfaces can still cause minor intravoxel signal dephasing and larger distortions in MS-IR-EPI than MP2RAGE. For T_1_ mapping of the temporal lobe near the sinus, a shorter echo time is required, which can be achieved by using parallel acquisition or more segments.

We expected the SNR per unit time in MS-IR-EPI to be higher than that of MP2RAGE. The MS-IR-EPI sequence is very time-efficient as the time between each inversion pulse is densely packed with excitation pulses and their acquisition windows, with minimal dead time compared with MP2RAGE, which needs dead time to allow the longitudinal magnetization to recover. In addition, the MS-IR-EPI acquisition uses 90° flip angle excitation pulses, allowing full use of the nuclear magnetization available, compared with the much lower flip angles used in 3D spoiled FLASH sequences (5° and 3° used in the first and second volume, respectively, in our MP2RAGE implementation). Prior to implementation of the study we optimized the RF pulses to optimize slice profile whilst minimizing side-bands, as imperfect thin slice profiles can leave some signal un-acquired in multi-slice 2D imaging. For the NIST spherical phantom, we measured higher SNR per unit time for T_1_ maps generated with MS-IR-EPI than with MP2RAGE. Use of a shorter TR improves the SNR per unit time, at the cost of accurate T_1_ estimation. When SMS acquisition was used, SNR was found to decrease only marginally.

In brain imaging, the SNR per unit time in GM was higher for MS-IR-EPI than in MP2RAGE T_1_ maps, without SMS acquisition, both with and without fat suppression, but with an SMS factor of two slices, and with fat suppression, it was slightly lower than that of MP2RAGE. Note that head motion between scans can result in errors in the estimation of the noise, measured as the difference between the two scans. In SMS acquisitions, SNR is degraded by the g-factor reconstruction noise, which varies considerable between scanners and vendors. In addition to the g-factor noise penalty, non-perfectly square slice profiles can cause signal loss within a slice due to partial saturation resulting from RF excitation meant for an adjacent slice Ideal slice profiles are harder to achieve for thinner slices, particularly when using SMS excitation. Another noise contribution is introduced by the fitting procedure required for the T_1_ mapping in MS-IR-EPI, compared with MP2RAGE which uses a look-up table. To reduce this source of noise, a look-up table could also be used to estimate T_1_ in MS-IR-EPI, as previous studies have shown (Renvall et al., 2016; Cohen and Polimeni, 2018).

Comparisons here were performed for a whole brain volume, where the SNR for 3D volumetric sequences such as MP2RAGE is optimal. By contrast, in applications for which only a partial brain volume is required, multi-slice sequences such as MS-IR-EPI incur no loss of SNR, and hence can provide better SNR per unit time.

Here, we have compared MP2RAGE and MS-IR-EPI acquisitions accelerated by parallel imaging techniques. However, recent advances in image reconstruction based on deep learning algorithms can be used to highly accelerate the acquisition in MP2RAGE (Lønning et al., 2019) and multi-shot EPI (Bilgic et al., 2019). Compressed sensing (Lustig et al., 2007) has also been applied to accelerate MP2RAGE acquisitions (Trotier et al., 2019; Mussard et al., 2020).

### 4.4 High resolution T_1_ mapping using MS-IR‑*EPI* and MP2RAGE

A key objective was to provide a sequence for T_1_ mapping which provides high SNR per unit time and sharp images to improve visualization of intra-cortical structures. T_1_ maps of the whole brain generated with MS-IR-EPI at the higher 0.5 mm isotropic resolution show good SNR and an exquisite level of detail. The acquisition time for 0.5 mm isotropic MS-IR-EPI data was under 20 min, considerably faster than the 60 min total acquisition time (30 min per hemisphere) reported for quantitative T_1_ maps of comparable SNR generated with 0.5 mm isotropic resolution using MP2RAGE at 7 T (Dinse et al., 2015). It should be highlighted that for SMS slice thicknesses of 0.5 mm, the pulse duration increases considerably to 9.7 ms, but SAR does not change with SMS factor or slice thickness (0.7 and 0.5 mm).

It has been suggested that a high spatial resolution, ideally to 0.3 mm, will be required to reliably distinguish the cortical areas solely based on intra-cortical features (Dinse et al., 2015). For this purpose, it is crucial that the nominal spatial resolution is not compromised by a poor PSF. But a slice profile for an ultra-thin slice (0.3 mm), is more difficult to achieve in 2D (as compared to 3D), due to the longer RF pulses to achieve a nominally rectangular slice profile and small side lobes to minimize slice-to-slice crosstalk. Here, we compared T_1_ maps generated from data acquired from the occipital pole of a volunteer subject with 0.35 × 0.35 × 0.7 mm^3^ resolution using MS-IR-EPI and MP2RAGE, taking a plane of section generally perpendicular to the calcarine sulcus. Although higher resolution is required to resolve the stria of Gennari in areas of high curvature, the cortical profiles across the striate cortex (calcarine sulcus) and extra-striate cortex from T_1_ maps generated using MS-IR-EPI are distinct, showing a clear dip in T_1_ indicating the presence of the Stria of Gennari, whereas this is not clearly evident for the T_1_ map generated using MP2RAGE (Fig. 8B). This finding is consistent with a narrower point spread function for the MS-IR-EPI acquisition compared to the MP2RAGE acquisition. In MP2RAGE the signal recovery during the acquisition window (1276.7 ms), which is relatively long with respect to T_1_ of GM and WM tissue, induces T_1_ blurring in the resulting image, which can be exacerbated by incorrectly chosen flip angles. In MS-IR-EPI there is less T_1_ relaxation during the short readout (BW=41 Hz/duration=24.4 ms) and hence less T_1_-induced blurring.

MS-IR-EPI images can also be prone to blurring in the phase encoding direction, associated with the relatively short T_2_*, due to the lines of k-space being acquired at different times. In our implementation this effect is minimal due to the short acquisition window used, resulting from a high readout bandwidth and low EPI factor (see Table 1), though the acquisition window may be significant relative to the T_2_* of tissue in areas of high susceptibility (due to iron, air, etc.). It is also worth noting that the use of motion correction across the individual MS-IR-EPI volumes may have introduced additional image blurring (uncorrected subject motion also introduces image blurring); however, even with these additional sources of blurring, the effective resolution of MS-IR-EPI was superior to that of MP2RAGE. The improved sharpness of MS-IR-EPI and efficiency (faster than MP2RAGE) highlight the feasibility of MS-IR-EPI for improving the spatial resolution of T_1_ mapping for the purpose of in-vivo cortical parcellation using myeloarchitecture.

For fully quantitative T_1_ analysis, a long TR (at least 5 s) is required. However, for the purpose of cortical parcellation, where the relative T_1_ profile across the cortex is of greatest interest, a shorter TR could be used to speed up the acquisition. Note, however, that a longer TR can be used to improve SNR, in place of signal averaging. For the purpose of cortical parcellation MS-IR-EPI presents an additional advantage as it also provides a S_0_ parameter fit, which is a map of proton density (with a small amount of T_2_* weighting), thus providing an additional contrast that could be used in conjunction with the T_1_ map to assist in cortical parcellation.

Although the aim of this study was to collect very high resolution T_1_ maps with excellent spatial fidelity, it should be noted that this MS-IR-EPI scheme can be adapted to acquire T_1_ maps which are distortion-matched to fMRI data acquisitions, by adjusting the acquisition bandwidth. This can provide 0.3 mm isotropic resolution T_1_ structural maps useful in cortical depth-dependant fMRI applications in fMRI space, especially important given the role of such maps in intra-cortical parcellation.

### 4.5 Considerations for ultra-high resolution T_1_ mapping

In order to obtain T_1_ maps with such spatial resolution as high as 0.3 mm isotropic, prospective motion correction methods, using either an optical tracking system (Zaitsev et al., 2006), NMR field probes (De Zanche et al., 2008), fat navigators (Versluis et al., 2010) or a combination of those, are highly desirable to overcome the increased sensitivity to very small movements likely to occur during the extended acquisition times. NMR field probes are sensitive to respiration in high magnetic fields (Boer et al., 2012; Vannesjo et al., 2015) and hence can correct artefacts due to both true motion and breathing. Since multi-shot EPI acquisitions are sensitive to dynamic B_0_-field fluctuations between shots, correction methods using NMR field probes can potentially improve the quality of multi-shot EPI data. A recent method has been proposed to improve image quality and quantitative metrics in multi-shot T_2_*-weighted imaging by retrospectively correcting for respiration induced B_0_-fluctuations with a respiratory trace (Vannesjo et al., 2019).

The slice profile of the slice-selective excitation pulses is another factor that requires attention when reducing the slice thickness for ultra-high spatial resolution imaging. Ideal rectangular profiles are harder to achieve for thin slices, which require long RF pulse durations, particularly when combined with SMS acquisitions. The use of high slice acceleration factors in SMS acquisitions can lead to RF pulses with high peak voltage and hence increased SAR. Several methods have been proposed to reduce SAR, for example by combining SMS pulses with parallel transmission, which also provides a more uniform excitation (Wu et al., 2013; Poser et al., 2014). Another way to reduce the maximum B_1_ amplitude is to increase the pulse length; for the SMS acquisitions described in this paper, the maximum B_1_ amplitude was limited to 5 mT, resulting in a modest increase in SAR (4%) for MS1/MS2 sequences, but larger (22%) for the 0.5 mm isotropic acquisition (MS3, SMS=3) with respect to the single slice excitation acquisition. The RF pulse duration (9.6 ms) required for 0.5 mm thick slices is significant longer than the pulse duration for a single slice acquisition (3.6 ms), leading to increase TE (14 instead of 12 ms) and making sharp slice profiles harder to achieve. In order to minimize interference from neighbouring slices due to non-perfectly square slice profiles, interleaved slice excitation schemes, which can be implemented with SMS imaging (Setsompop et al., 2012), can be employed. Alternatively, methods that acquire the image volume twice (with a spatial shift in the slice direction between acquisitions) and combine the data using an iterative super-resolution algorithm (Greenspan et al., 2002) can be explored to increase the resolution in the slice select direction with improved edge definition.

## 5 Conclusion

Accurate T_1_ quantification is feasible using our proposed novel image processing method, which corrects for the nontrivial magnetization transfer effects on T_1_ caused by the necessary spectrally-selective fat-suppression pulses. This implementation of MS-IR-EPI provides fast T_1_ maps which are sharper than those obtained with MP2RAGE. These methods could be applied to study quantitative measures of myelin in normally appearing white matter in clinical populations compared to the healthy brain (e.g. in multiple sclerosis (Al-Radaideh et al., 2015) and neuromyelitis optica (Chou et al., 2019)) to study intracortical demyelination processes in clinical conditions (e.g. in Alzheimer's disease (Luo et al., 2019) or multiple sclerosis (Mougin et al., 2016; Beck et al., 2018)), and to improve discrimination of functionally relevant cortical areas in human brain to correlate with fMRI studies.

## CRediT authorship contribution statement

**Rosa M. Sanchez Panchuelo:** Data curtion, Formal analysis, Methodology, Conceptualization, Funding acquisition, Writing – original draft. **Olivier Mougin:** Data curtion, Formal analysis, Methodology, Conceptualization, Writing – review & editing. **Robert Turner:** Methodology, Conceptualization, Writing – review & editing. **Susan T. Francis:** Methodology, Conceptualization, Funding acquisition, Writing – review & editing.

## Declaration of Competing Interest

None.

## Appendix Supplementary materials

Image, application 1
