# Supplementary material for: Quantitative T1 mapping using multi-slice multi-shot inversion recovery EPI
Source: Neuroimage. 2021 Jul 1;234:117976. doi: 10.1016/j.neuroimage.2021.117976 (PMC8204273; doi:10.1016/j.neuroimage.2021.117976)
Supplement: Supplementary file 1 [file mmc1.pdf]

Supplementary material

1. MonteCarlo simulations: determining optimal number of acquisitions

Monte Carlo simulations of multi-shot MS-IR-EPI sequence for acquisitions MS2 (TR-3.2s, 70 slices, 7T) and MS5 (TR=4.5s, 96 slices, 3T) are shown in Figure 1. For the MS2 acquisition (Fig.1A), the standard deviation across the slices shows that there is a variation of the fitted T_1 value across slices when using 3 and 4 TIs for WM, whereas in GM the variation is only obvious when using only 3 TIs. For the MS5 3T acquisition (Fig.1B), simulations show that when using 8 TIs the variation is small for both GM and WM.

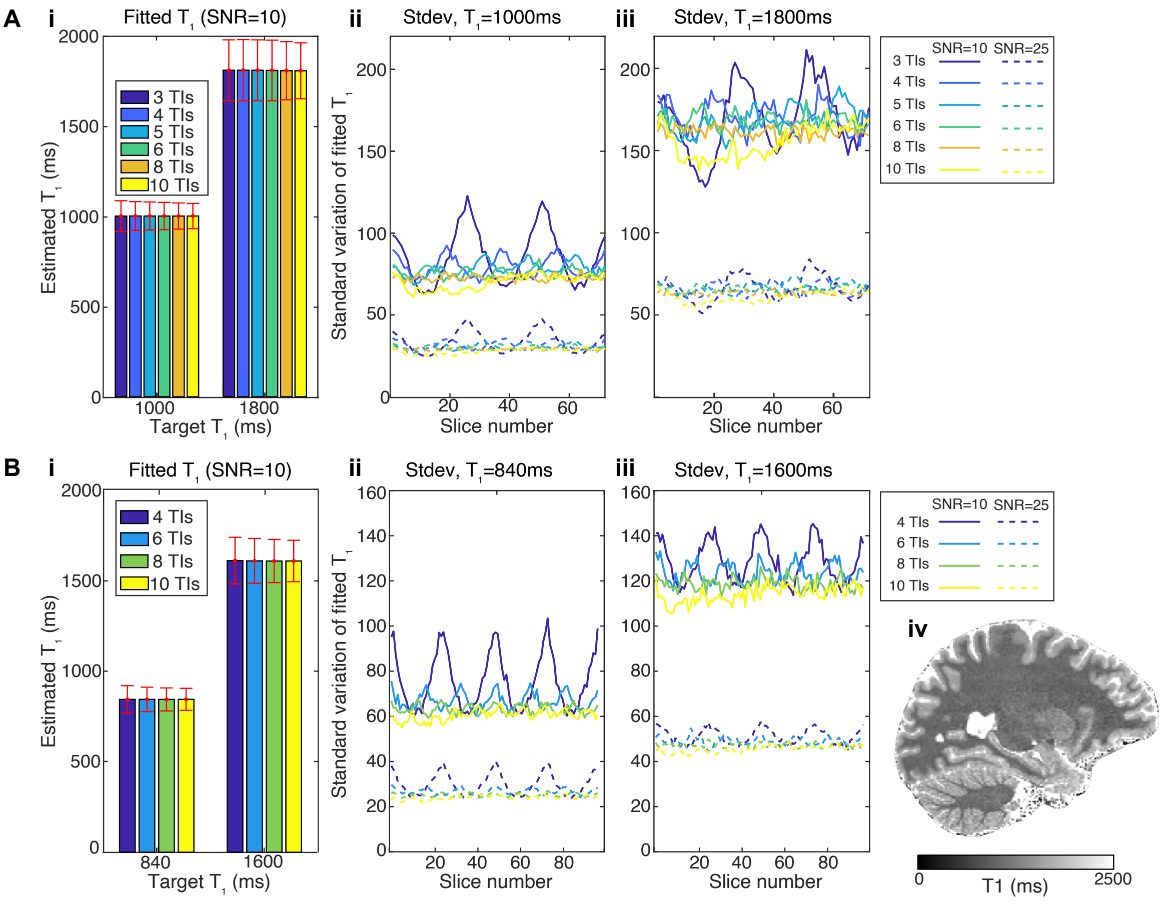

Figure 1: *Monte Carlo simulations of multi-shot multi-slice inversion recovery EPI sequence for acquisitions with (A) TR=3.2s (72 slices, 7T) and (B) TR=4.5s (96 slices, 3T) showing the effect of number of acquisition/TIs (3,4,5,6,8,10) on fitted T_1 parameters for a target T_1 values typical of white and grey matter at 7T (A) and 3T (B). Gaussian distributed random noise (with SNR levels of 10 and 25) was added to the complex simulated data (with 1024 samples) which was fit, as for the experimental data, to obtain mean and standard deviation of fitted T_1 values. (A) (i) The fitted T_1 (mean across slices), for T_1 targets of 1000 and 1800, remains constant but the standard deviation (error bars) decreases with increasing number of TIs. (ii) Standard deviation plot of the fitted T_1-values across slices for WM and (iii) for GM for SNR levels of 10 (solid line) and 25 (dash lines). (B) (i) Fitted T_1 for targets of 840 and 1600ms, representative of WM and GM at 3T. Standard deviation of the fitted T_1-values for (ii) WM and (iii) GM across each of 96 slices. (iii) Sagittal reconstruction of the axial T_1-maps derived using 8 equally spaced TIs from a MS-IR-EPI acquisition with 1mm isotropic resolution. A SPIR fat suppression pulse with a low flip angle (FA=12º) was used to supress the fat artefact.*

2. Adiabatic inversion pulse: effect of efficiency on the accuracy of T_1

We simulated the error measured on T_1 for three target values (1200, 1600 and 2000 ms) for different efficiency levels of the adiabatic inversion pulse (Figure 2). The red line shows no error for a perfect inversion (efficiency=1, 180°) irrespectively of TR. When the inversion pulse is not perfect, T_1 is overestimated but this error decreases with increasing TR: for efficiency=0.9 (corresponding to a flip angle of 162°) the error for a target T_1 of 1200ms (~WM at 7T) decreases from 20ms for a TR=1s to less than 10ms for TR>3s. Although this error increases for longer T_1 target values, the error for target T_1 of 2000ms remains low (10.7ms) for the TR (5s) used in our experiments.

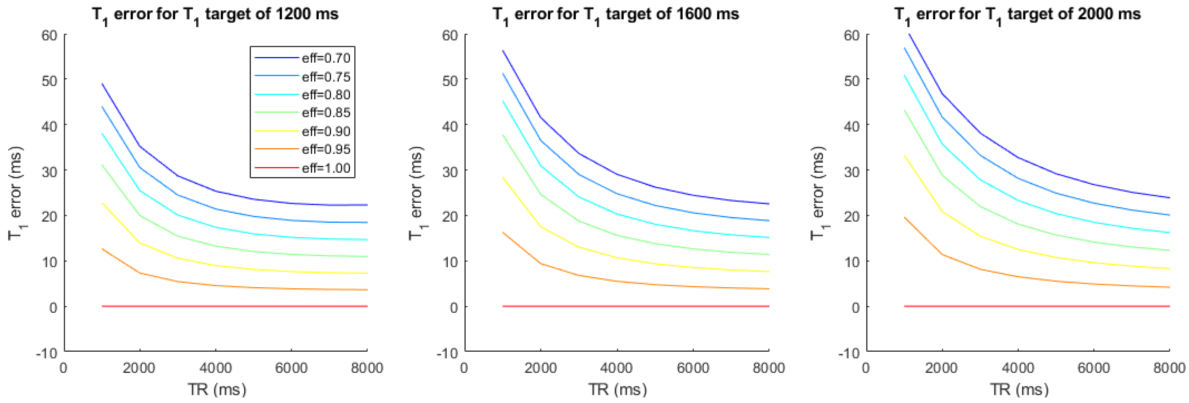

Figure 2: *Simulations of the expected error in T_1 measurements for target T_1 values of 1200ms (left), 1600ms (middle) and 2000ms (right) as a function of TR for different levels of efficiency of the adiabatic inversion pulse, ranging from 1 (perfect inversion: 180° flip angle) to 0.7 (126° flip angle).*

3.Measurements of ISMRM/NIST system T_1 and T_2-spheres.

Previous work using the ISMRM/NIST system T_1-spheres had showed that the DREAM B_1+ mapping sequence is only accurate for T_1 values larger than approximately 300ms (Kato et al., 2019). Our B_1-field measurements with the DREAM sequence inside each T_1-sphere showed that this was also the case at 7T (Fig.3A). The B_1-field measured inside spheres T_1_7 to T_1_14 at 7T was reduced from the nominal B_1 by more than 50%, in spheres T_1_5 and T_1_6 the value of B_1 was reduced by 67 and 58% respectively, whilst for spheres T_1_1 to T_1_4 the B_1 was above 80% of its nominal value.

One consideration for T_1-mapping with the MP2RAGE sequence is that the MP2RAGE signal intensity is not monotonic for the chosen acquisition parameters at 7T (which was based on the sequence described by Haast et al. 2018). Since the MP2RAGE signal intensity values in the lookup table do not have a discrete one-to-one correspondence for low T_1, some spheres may comprise voxels which are assigned T_1 values on either side of the 540ms peak at 7T (Fig.3C), as it is the case for spheres T_1_5 and T_1_6 (~400 and 290ms respectively) where some voxels have been clearly misclassified, as shown by the noisy map within these spheres in Fig.3B. Histograms of MP2RAGE T_1-values (Fig.3D) show two peaks for T_1_5 and T_1_6, but also for T_1_4 (this was also the case for T_2 spheres T_2_7, T_2_8 and T_2_9). Whenever two histogram

peaks were present, a cut-off T_1-value was applied to measure T_1-values only across the voxels contributing to the correct peak (based on the knowledge of expected T_1 reference value).

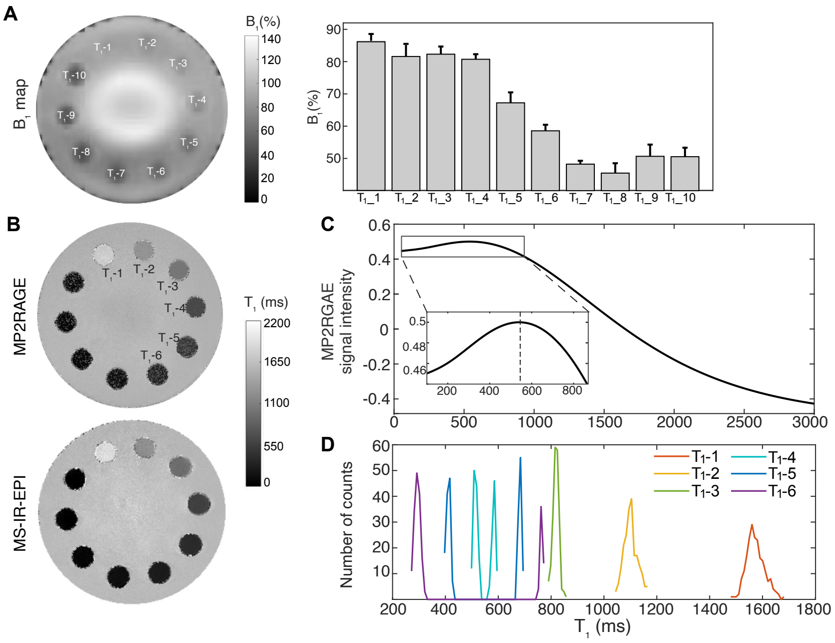

Figure 3: *Results for ISMRM/NIST system T_1 phantom at 7T. (A) B_1-map (%) collected using a DREAM and associated bar plot of B_1 in each T_1 sphere. Error bars represent standard deviation across voxels. Note the B_1 in spheres T_1_7 to T_1_10 falls below 50%. (B) T_1 maps obtained with the MP2RAGE (top) and MS-IR-EPI (bottom) scheme. (C) Look-up table of the MP2RAGE signal intensity versus T_1. The MP2RAGE signal intensity increases with increasing T_1 values until ~540ms (dash line in inset) and then decreases with increasing T_1 values, hence at low T_1 there is not a discrete solution. As a result, spheres T_1_5 and T_1_6 have a MP2RAGE signal intensity which matches two different T_1 values (one at either side of 540ms peak) and have been miss-classified. (D) Histograms of MP2RAGE T_1-values for spheres T_1_1 to T_1_6. Notice that for spheres T_1_1, T_1_2 and T_1_3 (red, tallow and green respectively) there is a single peak, whereas for spheres T_1_4, T_1_5 and T_1_6 (cyan, blue and purple respectively) there are two peaks.*

Details of mean T_1 values (across sessions) measured in NIST T_1 spheres at 3T and 7T for each T_1-mapping method, along with their respective $NiCl_2$ concentration and reference 3T T_1 values provided by NIST are provided in Table 1. Note a large variation of measured T_1-value is apparent in the T_1_3 sphere across time (but consistent values were measured with all T_1-mapping methods for this sphere within a scan session); T_1-values measured using the standard single-slice IR sequence were 966±17ms and 820±19ms at 3T and 7T respectively for session 1, and 1414±9ms and 1227±9ms at 3T and 7T respectively in the scan session 9 months later. Hence, for the T_1_3 sphere, the accuracy was evaluated based on the first measurement alone, while the coefficient of variation (CoV) was evaluated on the last four measurements acquired within a 10 day window.

Table 2 contains the corresponding information for the NIST $MnCl_2$-doped T_2 spheres. Note that the reference T_1 values for spheres T2_1 and T2_5 are reported with respect to known deviations of the $MnCl_2$ concentration due to an error in the manufacturing process.

***Table 1**: Mean T_1 values obtained in each NIST T_1-sphere for each T_1-mapping sequence. Mean across three measurements at 3T and across six measurements at 7T. For sphere T_1_3, mean across voxels of a single measurement.*

Sphere	$NiCl_2$	T_1 at 3T (ms)				T_1 at 7T (ms)		
Number	concentration	Reference	IR-TSE	MS-IR-EPI	MP2RAGE	IR-EPI	MS-IR-EPI	MP2RAGE
T_1_1	0.299	1989±1.0	2015±30	2039±49	1944±86	1760±32	1768±44	1615±58
T_1_2	0.623	1454±2.5	1386±16	1421±14	1415±63	1171±23	1194±22	1144±27
T_1_3	1.0720	984.1±0.33	966±7	1034±47	985±18	820±19	835±17	818±12
T_1_4	1.720	706±1.5	694±5	711±7	650±32	555±5	567±8	588±6
T_1_5	2.617	496.7±0.41	489±4	508±4	411±61	388±2	407±18	406±7
T_1_6	3.912	351.5±0.91	364±4	364±4	372±29	406±7	272±4	289±13

***Table 2**: Mean T_1 values obtained in each NIST T_2-sphere for each T_1-mapping sequence. Reference values (batch #2) for 3T at 20°C. Mean across two measurements at 3T and across four measurements at 7T. No result shown for sphere T_2_10 for MP2RAGE at 7T as look-up table was constrained to T_1 >300ms.*

Sphere	$MnCl_2$	T_1 at 3T (ms)				T_1 at 7T (ms)		
Number	concentration	Reference	IR-TSE	MS-IR-EPI	MP2RAGE	IR-EPI	MS-IR-EPI	MP2RAGE
T_2_1	0.0108	3025±76	2722±6	2795±47	2299±1	2880±78	2972±104	2460±34
T_2_2	0.0181	2186±55	2030±13	2059±86	2081±45	2085±50	2157±47	1937±106
T_2_3	0.0282	1901±48	1739±16	1806±52	1840±13	1779±33	1840±42	1751±115
T_2_4	0.0434	1550±39	1399±28	1464±38	1515±29	1464±39	1507±42	1422±78
T_2_5	0.0626	1511±38	1363±27	1415±44	1479±34	1421±36	1451±49	1385±79
T_2_6	0.0934	1026±26	916±17	972±28	961±30	937±32	973±31	937±30
T_2_7	0.1353	805±20	713±12	768±19	690±57	721±18	765±16	755±14
T_2_8	0.193	600±15	512±11	575±12	473±25	531±11	569±18	575±31
T_2_9	0.2768	431±11	352±7	401±4	341±9	365±4	399±24	428±10
T_2_10	0.4276	293±7	233±4	267±10	306±12	237±14	281±29	-

4. Alternative method for correction of MT effects from the T_1 quantification

An alternative method to compute the *b/a* parameter maps is to reduce the amount of fat suppression pulses, rather than varying the FA of the SPIR fat suppression pulse. On one subject we performed two acquisitions using SPIR fat suppression pulse with FA=90°, one in which the SPIR pulse was applied prior to each RF acquisition (all) and one in which a SPIR pulse was applied prior to every alternate RF acquisition (alternate), hence using 50% of the number of FS pulses. Additional acquisitions were performed with a set of SPIR pulses of nominal FA=[0°, 30°,45°,60°,78°] applied prior to each RF acquisition. Figure 4 shows comparison of voxel wise correction methods in the absence of a B_1-map. Notice similar histogram for data with FS flip angle of 45° (yellow) and data with alternate FS pulses of 90° flip angle (grey). The b/a parameter maps were spatially smoothed by a Gaussian kernel with a full width at half maximum (FWHM) of 2mm prior to correction to improve the SNR of the corrected R_1-maps. Notice that the corrected histograms are wider (Fig4B.ii,iii) when only two data sets (instead of 4) are used for correction. Figure 4C shows original and corrected R_1-maps for each method. The red arrowhead indicates a region of hyperintensity in the uncorrected R_1-maps (due to spectrally selective FS pulse causing attenuation of the proton signal), which is more evident for FS pulses with higher flip angles. The

corrected R_1-maps however are homogeneous across the brain as the spatial variations are reflected (and corrected) by the fitted voxelwise *b/a* parameter map.

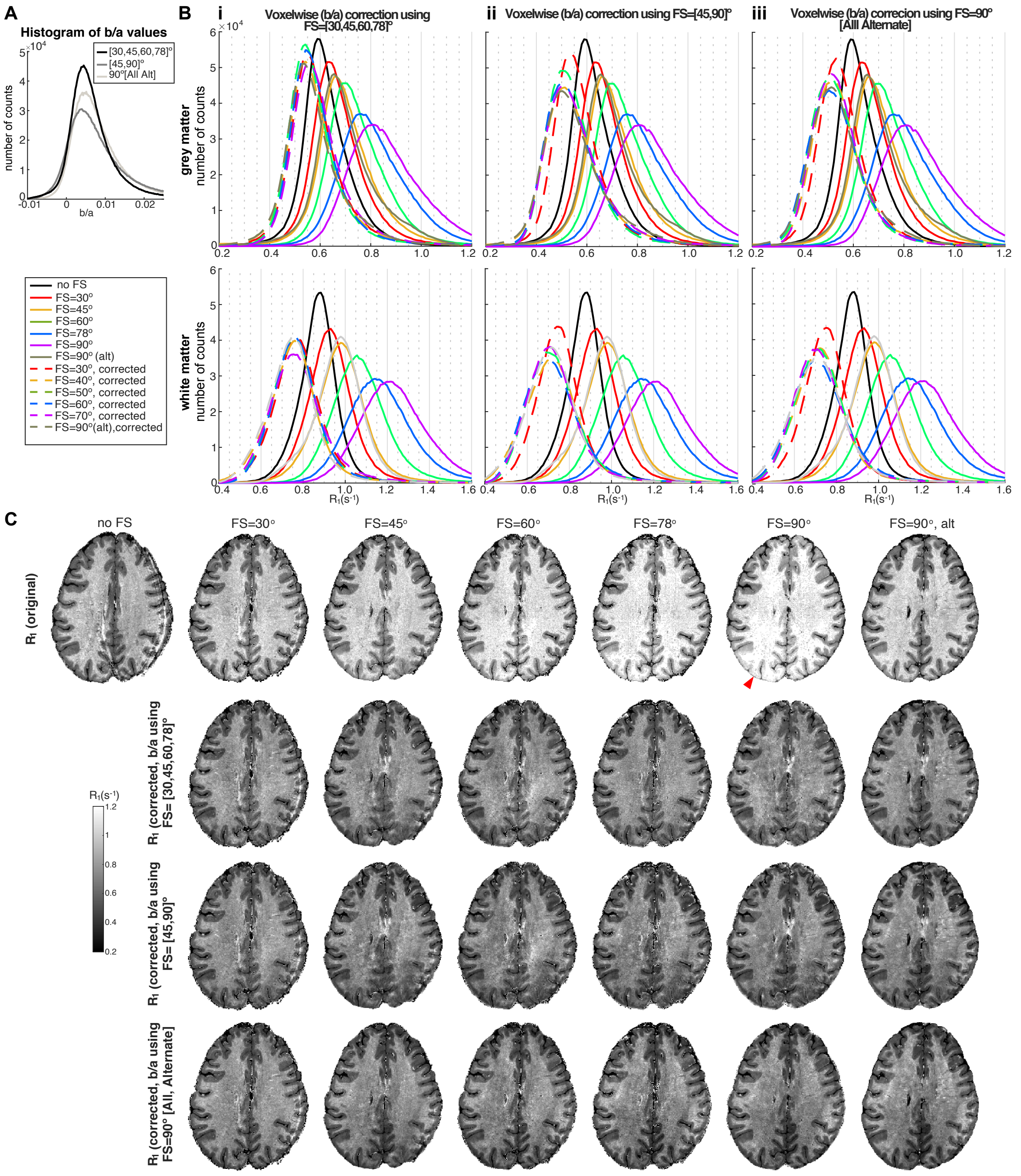

Figure 4: *(A) Histogram of b/a values obtained by linear regression of R_1 versus SPIR flip angle when considering datasets comprising 4 FS flip angles of 30°, 45°, 60°, and 78° (black line), 2 FS flip angles of 45° and 90° (dark grey), data acquired with FS flip angle of 90° either prior to all acquisitions or prior to every other acquisition [All Alternate]. (B) R_1 histograms for grey matter (top) and white matter (bottom) from data acquired with different levels of fat suppression (solid colour lines) compared to data acquired with no fat suppression (black line) and corresponding R_1 histograms after correction (dashed lines) using b/a parameter maps generated from (i) 4 FS flip angles [30°, 45°, 60°, and 78°], (ii) 2 FS flip angles [45° and 90°] and (iii) FS flip angle of 90° prior to all or alternate excitation pulses. (C) Original R_1-maps (first row) shown for the different SPIR flip angles and corresponding R_1-maps after correction with b/a map derived from the three different methods.*

5. *b/a* parameter estimation results for subjects 4 and 5 (section 3.5)

Five subjects participate in the experiment to compare MS-IR-EPI with single slice IR-EPI in a single slice and MP2RAGE across the whole brain. Subject specific *b/a* parameter values were available for subjects 1, 2 and 3, who had participated in the experiment to model the impact of FS pulses (Section 2.2.1). For subjects 4 and 5, the *b/a*-parameter map was estimated from two data sets acquired with SPIR flip angles of 40° and 70°. Figure 5 plots histograms of b/a parameter values measured for subjects 3 and 4. The *b/a* parameter for the global correction was computed from the two Gaussian mixture fit, using the mode (peak) of the highest amplitude Gaussian (0.0043 and 0.0042 for subjects 3 and 4 respectively).

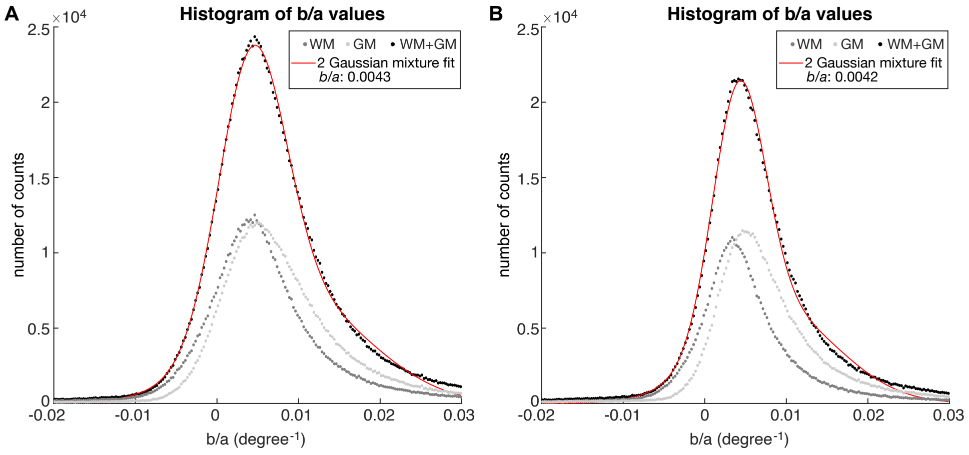

Figure 5: *Histogram of b/a parameter values obtained by linear regression of R1 versus SPIR FS flip angle for GM tissue (dark grey), WM tissue (light grey) and total (GM+WM) tissue (black) for (A) subject 4 and (B) subject 5. Red line: two Gaussian mixture fit to the (GM+WM) data.*

6. LCPA denoising of high spatial resolution data

Complex denoising was performed using a local complex principal component analysis (LCPCA) technique (Bazin et al., 2019) which has been shown to efficiently improve SNR of MR images acquired at the lower limit of SNR. Figure 6A shows the effect of de-noising for an example slice acquired at very high spatial resolution (0.35x0.35x0.7mm^3) with MS-IR-EPI (acquired at inversion time of 495ms) using the surface coil. The SNR improvement is very clear, particularly for the cerebellum where SNR was very low due to the positioning of the surface coil. The SNR improvement is not so dramatic for the derived T_1-map (Figure 8 in main manuscript), probably due to the SNR improvement when combining the individual acquisitions (n=15) to compute the T_1-map. Cortical profiles extracted from the T_1 and M_0 maps derived before and after de-noising are very similar (Fig. 6B).

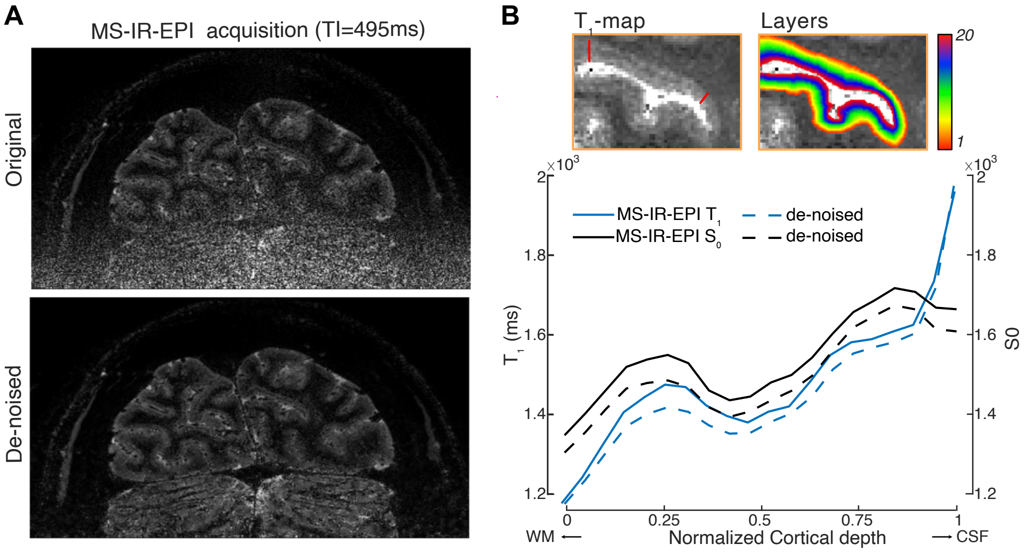

Figure 6: *(A) High spatial resolution (0.35x0.35x0.7mm^3) MS-IR-EPI data (TR=3.2s, TE=20ms, EPI factor= 13, 35 slices, offsets=[0,7,14,21,28], 3 averages, 11min 8s total acquisition time) for an example slice acquired with TI=495ms before (top) and after (bottom) complex de-noising. (B) Cortical profiles for original (solid lines) and de-noised (dash lines) fitted T_1 (blue) and M_0 (black) maps: mean across profiles between the two boundaries indicated by the red lines in the T_1-map inset (top left). Cortical depths derived using the equi-volume approach are shown in the top left corner.*
